# Unveiling the inflammatory messengers after intracerebral hemorrhage: the crosstalk between peripheral NETs and microglia

**DOI:** 10.3389/fimmu.2025.1643524

**Published:** 2025-09-17

**Authors:** Shanshan Zhang, Ziqi Jin, Li Jiang, Yibin Zhang, Tong Wu, Peng Xu, Yabin Cui, Dongmei Zhang, Jing Lu

**Affiliations:** ^1^ Department of Traditional Chinese Internal Medicine, Changchun University of Chinese Medicine, Changchun, Jilin, China; ^2^ Department of Encephalopathy, The Affiliated Hospital to Changchun University of Chinese Medicine, Changchun, Jilin, China; ^3^ Research Center of Traditional Chinese Medicine, The Affiliated Hospital to Changchun University of Chinese Medicine, Changchun, Jilin, China

**Keywords:** intracerebral hemorrhage, neutrophil extracellular traps, microglia, central and peripheral immune systems, mechanism analysis

## Abstract

Intracerebral hemorrhage (ICH), a common neurological disorder with a high rate of disability, involves complex immunoinflammatory mechanisms, particularly those related to secondary inflammatory injury. Neutrophils, as the earliest subtype of leukocytes recruited after stroke, play a pivotal role in secondary brain injury. Traditionally, neutrophils were thought to mediate tissue damage primarily via phagocytosis, chemotaxis, and degranulation. However, recent studies have shown that neutrophils also contribute to the pathogenesis of intracerebral hemorrhage by releasing neutrophil extracellular traps (NETs), which exacerbate blood-brain barrier disruption, amplify local inflammy -30ation, and promote neuronal injury. This review systematically examines the interactions between the central and peripheral immune systems following ICH. It focuses on the bidirectional regulatory relationship between microglia and neutrophils, and their coordinated roles in inflammation, blood-brain barrier disruption, neurological dysfunction, and cognitive impairment. In addition, this review summarizes recent potential therapeutic strategies targeting the formation and clearance of NETs, including peptidylarginine deiminase 4 inhibitors, reactive oxygen species inhibitors, histone inhibitors, and DNases. These interventions may offer theoretical insights into novel therapeutic targets for mitigating secondary injury following ICH.

## 1 Introduction

Stroke ranks among the foremost causes of mortality and disability globally, with its prevalence steadily rising in recent years, particularly in developing countries and aging populations. According to the latest statistics, in 2018, the mortality rate for cerebrovascular diseases in China was 149.49 per 100,000, with 1.57 million deaths (1). By 2020, the mortality rate for strokes had increased to 343.4 per 100,000 (2), with approximately 2.3 million deaths (3). In Europe and the United States, over 1.1 million and 790,000 new stroke cases (4), respectively, are reported annually, with a substantial proportion occurring in individuals with a history of stroke (5). The combination of high incidence, mortality, and disability rates has made stroke a pressing global public health challenge (6).

ICH, one of the most devastating type of stroke, encompasses parenchymal hemorrhage and subarachnoid hemorrhage (SAH), accounting for 15-30% of all stroke cases (7). The one-month mortality rate following onset can be as high as 40% (8). Currently, there are no effective neuroprotective treatments that significantly improve functional outcomes after ICH. Therefore, elucidating the pathological mechanisms underlying secondary injury following ICH and identifying potential therapeutic targets have become key priorities in both clinical and basic research. Following ICH, alongside the primary damage inflicted by the hematoma, secondary injuries such as neuroinflammation, immune cell infiltration, cerebral edema, and oxidative stress also occur (9–13). Among these, neuroinflammation is widely recognized as a central component of secondary injury (14).

Microglia, the resident immune cells of the central nervous system (CNS), promptly react to injury signals subsequent to ICH. Their activation states and polarization phenotypes play a decisive role in determining the extent of neuroinflammation and subsequent tissue repair (15, 16). Meanwhile, accumulating evidence indicates that involvement of the peripheral immune system—particularly the early infiltration of neutrophils into the brain—plays a critical role in amplifying the inflammatory response (17, 18).

During the immune response, neutrophils are rapidly recruited and activated, releasing nuclear and granular contents that form extensive web-like DNA structures known as NETs (19–21). NETs are composed of extracellular double-stranded DNA combined with various components, including histones, neutrophil elastase, myeloperoxidase (MPO), and cathepsins (22). In addition to their role in antimicrobial defense, NETs have emerged as a novel mechanism contributing to neuroinflammation and tissue damage (23). Studies have shown that NETs not only disrupt the blood-brain barrier (BBB) and increase its permeability but also promote the release of inflammatory mediators, thereby amplifying the local immune response (24, 25). Although the independent roles of microglia and neutrophils in the pathophysiology of ICH have been extensively reported, their interactions, particularly the molecular mechanisms linking NETs with central immune cells, remain poorly understood. Notably, most current studies focus on ischemic brain injury, leaving the immune response patterns specific to hemorrhagic stroke relatively underexplored.

This review systematically summarizes the interactions between the central and peripheral immune systems following ICH. It focuses on the signaling crosstalk and pathological synergy between microglia and neutrophils, particularly the bidirectional regulatory role of neutrophil NETs in this process. By outlining recent advances in this field, we aim to provide a theoretical foundation and reference for understanding the mechanisms of neuroinflammation in ICH, identifying potential biomarkers, and developing targeted therapeutic strategies.

## 2 Immune response following intracerebral hemorrhage

### 2.1 Interactions between the central and peripheral immune systems

**Figure 1:**
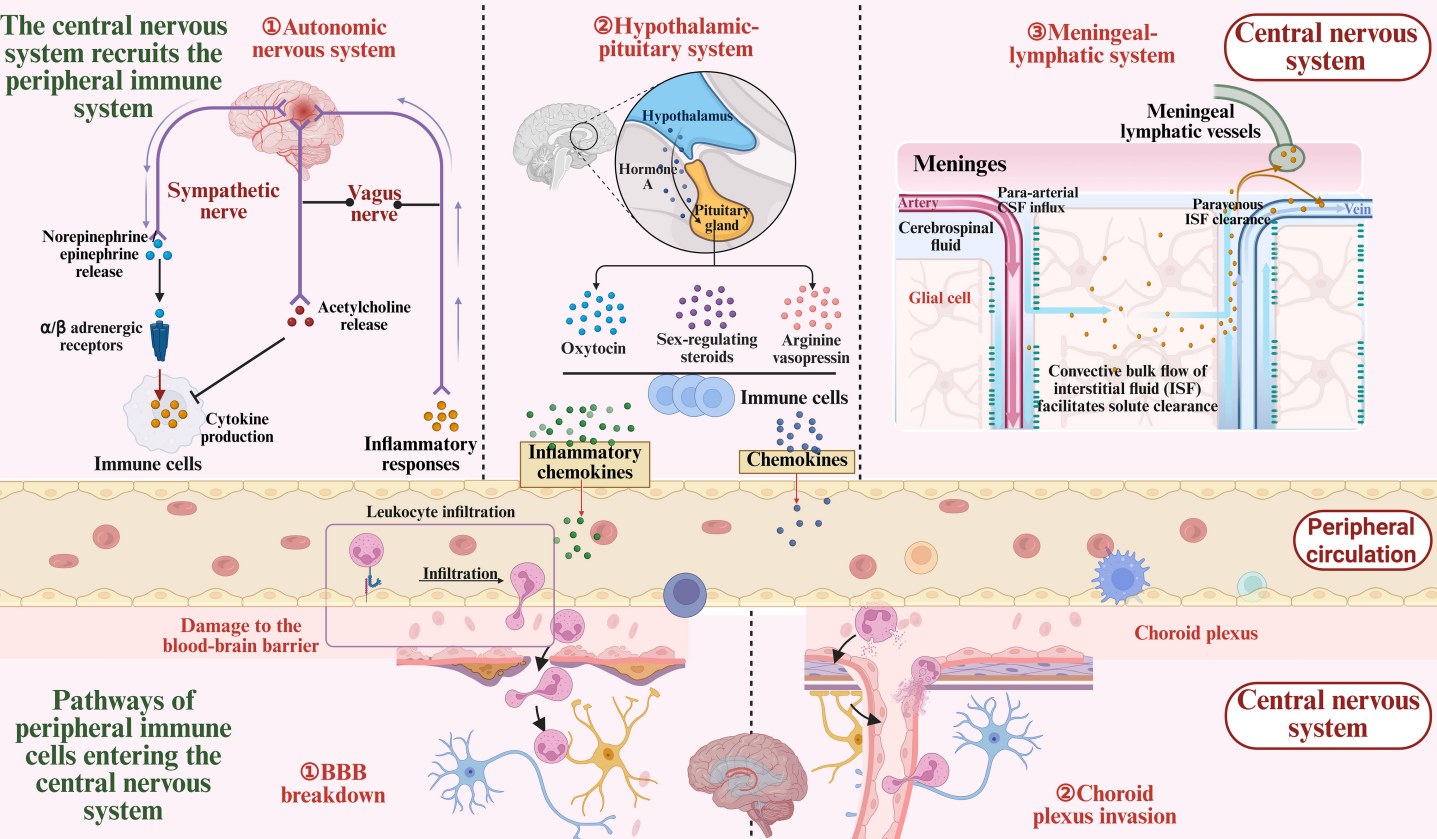
Bidirectional communication between the central and peripheral immune systems after ICH. Following intracerebral hemorrhage, the central nervous system (CNS) rapidly responds by engaging in bidirectional communication with the peripheral immune system via the autonomic nervous system, neuroendocrine system, andmeningeal lymphatic vasculature, while releasing signaling molecules such as inflammatory cytokines and chemokines. Meanwhile, peripheral immune cells infiltrate the CNS through the compromised blood-brain barrier and the choroid plexus, contributing to the local inflammatory response.

Due to its antigen-induced immune tolerance and the physical isolation provided by the BBB and the blood-cerebrospinal fluid barrier, the CNS has long been considered an immune-privileged site. The presence of peripheral immune components within the central CNS was traditionally considered a pathological feature under healthy conditions. However, research over the past two decades has gradually challenged this view (26). Growing evidence indicates that the CNS and the peripheral immune system are not entirely distinct entities, but instead form an interactive network through complex crosstalk (27, 28). As illustrated in **Figure 1**. The immune system maintains immunosurveillance homeostasis by detecting not only pathogenic signals but also cues from damaged tissues, particularly in sterile injuries such as traumatic brain injury (TBI), spinal cord injury, and ICH (29–31). Consequently, following ICH, the peripheral immune system performs a vital function in secondary injury and directly influences long-term outcomes.

ICH occurs due to vascular rupture, resulting in hematoma formation within the brain. The primary injury is characterized by mechanical compression of brain tissue, including elevated intracranial pressure and brain herniation. The subsequent detrimental effects are termed secondary brain injury, which encompasses neuroinflammation, cerebral edema, BBB disruption, and other pathological alterations. Inflammation is one of the earliest defense responses following ICH, but it also contributes to the exacerbation of brain injury. Both central nervous system and peripheral immune cells play critical roles in orchestrating the inflammatory response surrounding the hematoma (32). The release of inflammatory mediators, activation and migration of immune cells, tissue destruction, cerebral edema formation, and neuronal repair together constitute this complex physiological and pathological process (33). In fact, the initial inflammatory response in the CNS may represent a component of the innate immune response, marked by the generation of damage-associated molecular patterns (DAMPs). These molecules activate resident innate immune cells, including microglia and astrocytes, which subsequently phagocytose dead cells and release cytokines and chemokines to initiate neuroinflammation. More precisely, following injury, the CNS activates and recruits components of the peripheral immune system through multiple pathways, including the autonomic nervous system, the neuroendocrine system, and the meningeal lymphatic vasculature. These immune components can access the CNS via various routes such as the BBB, where they interact with resident CNS cells to exert both detrimental and beneficial effects (34, 35). As illustrated in **Figure 1**.

The sympathetic nervous system is integral to the stress response after stroke (36). It exerts its effects by releasing norepinephrine and epinephrine, which activate α- and β-adrenergic receptors expressed on immune cells. The expression levels of these receptors are closely associated with the activation state and functional status of immune cells (37, 38). Through activation of these receptors, the CNS can modulate immune cell migration and cytokine secretion (39–41). However, these effects can be both beneficial and detrimental (42). For example, activation of β-adrenergic receptors can enhance CD4^+^ T cell proliferation and cytokine production, but may also suppress macrophage responses to lipopolysaccharide, thereby increasing the complexity of the immune response (43–45). In addition, the vagus nerve can sense inflammation via its afferent arc and suppress immune responses by releasing acetylcholine, particularly by inhibiting macrophage-derived TNF-α, thus mitigating inflammation (46–48).

More profound immune regulation is also mediated by the neuroendocrine system. The hypothalamic-pituitary axis modulates immune responses not only through the release of oxytocin and arginine vasopressin (AVP) (49–51), but also by regulating the immunological functions of sex steroids such as testosterone and estrogen, thereby influencing immune cell activity (52, 53). Studies have shown that oxytocin and AVP exert anti-inflammatory effects (54–57), whereas sex hormones exhibit dual roles in either suppressing or enhancing immune responses (53). In addition, thyroid hormones play an important role in immune responses by promoting lymphocyte proliferation and immunoactivation, a phenomenon associated with the reduced immune function observed after thyroidectomy (58, 59). Nonetheless, the precise mechanisms and regulatory factors underlying these neuroendocrine effects remain to be elucidated.

The meningeal lymphatic system also plays a critical role in the immune response following stroke (60). Recent studies have identified functional lymphatic vessels in the meninges that connect to the peripheral immune system, facilitating the clearance of immune waste from brain tissue and guiding immune cell trafficking (61–63). This process is particularly important after stroke. Meningeal lymphatic vessels not only mediate the transport of immune cells from the CNS meninges and cerebrospinal fluid (60), but also direct neuron-specific antigens released after injury to the peripheral immune system, thereby initiating immune responses. For example, in patients with acute stroke, neuron-derived antigens have been detected in deep cervical lymph nodes, where they are presented to T cells by antigen-presenting cells, triggering autoimmune responses (64, 65). Following ICH, NETs induce damage to lymphatic endothelial cells and promote lymphatic thrombosis through CX3CR1 signaling, ultimately contributing to secondary brain injuries such as hydrocephalus (66).

Peripheral immune cells access the CNS primarily via two routes: the classical BBB pathway and the choroid plexus pathway. In the early phase after injury, immune cells are recruited to the cerebral vasculature and, through interactions with endothelial cells, traverse the BBB into the brain parenchyma via adhesion molecules (67–70). Following intracerebral hemorrhage, increased BBB permeability facilitates immune cell infiltration (71), which drives the progression of the inflammatory response. Research indicates that this process is initiated within minutes after injury, as immune cells gradually infiltrate the central nervous system through vascular inflammation and endothelial activation, leading to the amplification of the inflammatory response. The choroid plexus, serving as a critical interface between the CNS and the peripheral immune system, also plays a significant role in post-stroke immune responses (72, 73). The choroid plexus is not only responsible for cerebrospinal fluid production but also contributes to the circulation and trafficking of immune cells. Studies have shown that peripheral immune cells can enter the ventricles via the choroid plexus and subsequently infiltrate the brain parenchyma, thereby modulating the immune status of the CNS. This finding highlights an alternative pathway for immune cell entry into the CNS (74), particularly in the context of primary central nervous system injury, where the peripheral immune system contributes to the secondary immune response through the choroid plexus.

In summary, stroke-induced peripheral immune responses involve multiple complex mechanisms, including regulation by the autonomic nervous system, neuroendocrine modulation, immune cell trafficking through meningeal lymphatic vessels, and alterations in BBB permeability. This intricate immune process affects not only the acute phase of stroke but may also significantly influence long-term neural repair and recovery. Therefore, a deeper understanding of these mechanisms is essential for the formulation of effective immunomodulatory strategies.

### 2.2 Immunological functions of microglia in the central nervous system

Microglia are among the most important immune cells in the central nervous system, comprising approximately 5% to 10% of the total CNS cell population (75). They not only perform immune surveillance by secreting cytokines but also exhibit phagocytic activity (76), enabling them to clear dead cells and cellular debris. As such, they are often referred to as the “macrophages of the brain.” (77). Following stroke, microglia play a pivotal role in the central nervous system response. They are involved not only in the immune response to brain injury but also in the regulation of neuroinflammation, as well as in neural repair and remodeling.

**Figure 2:**
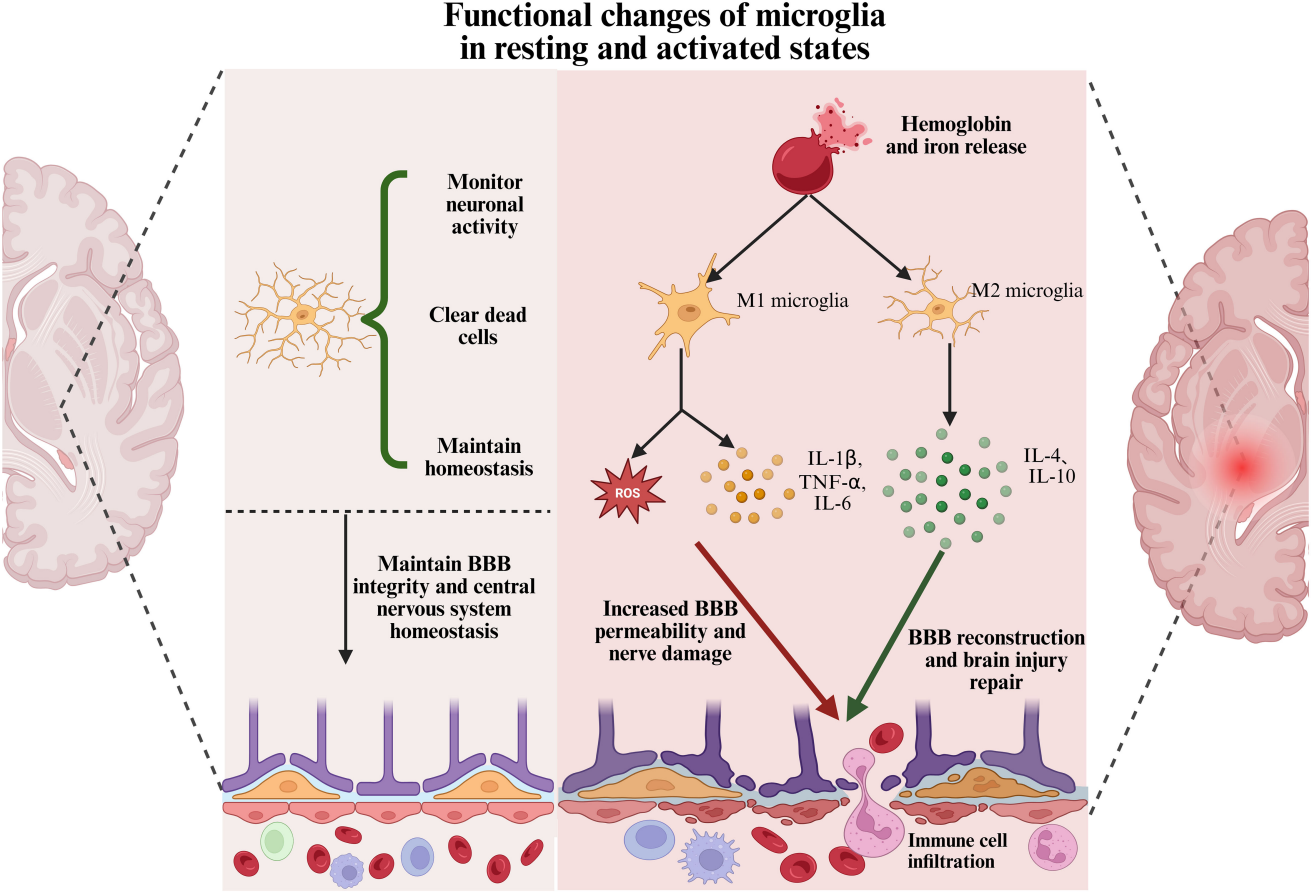
Functional changes of microglia in resting and activated states. In healthy brain tissue, microglia exist in a resting state with a ramified morphology. They monitor neuronal activity, clear apoptotic cells, and maintain central nervous system homeostasis. After ICH, microglia are activated by damage-associated molecules such as hemoglobin and iron. They adopt a pro-inflammatory M1 phenotype and release cytokines, including IL-1β, TNF-α, IL-6, and reactive oxygen species. This contributes to BBB disruption, immune cell infiltration, and neuronal damage. Subsequently, microglia transition to an anti-inflammatory M2 phenotype, secreting factors such as IL-4 and IL-10. This shift supports blood-brain barrier repair and promotes neurological recovery.

In healthy brain tissue, microglia exist in a “resting” state, primarily maintaining homeostasis by monitoring the microenvironment and clearing dead cells (15). However, following ICH, the release of hemoglobin and iron from the hematoma induces microglial activation (78). Activated microglia then migrate to the injury site and shift toward a pro-inflammatory M1 phenotype, releasing large amounts of cytokines, chemokines, and oxidative molecules, such as IL-1β, IL-6, and TNF-α (79, 80). The excessive immune response of microglia following ICH is not only neurotoxic but also exacerbates brain injury by increasing BBB permeability and promoting the infiltration of peripheral immune cells (81, 82). Studies have shown that excessively activated microglia disrupt the BBB by releasing large amounts of pro-inflammatory cytokines and matrix metalloproteinases (MMPs), thereby exacerbating damage to the surrounding neural tissue (82). Moreover, the release of these inflammatory mediators can induce the polarization of astrocytes into the neurotoxic A1 phenotype, thereby amplifying the inflammatory response. These pro-inflammatory cytokines also promote the infiltration of additional immune cells, creating a vicious cycle that further exacerbates inflammation and neuronal injury (80). As illustrated in **Figure 2**. Therefore, modulating the activation state of microglia is critical for improving stroke outcomes and guiding therapeutic interventions.

While microglial activation enhances the local immune response, it also exerts neuroprotective effects by phagocytosing damaged cells and cellular debris to clear the injury site (83). Within seven days following ICH, microglia transition from a pro-inflammatory M1 phenotype to an anti-inflammatory M2 phenotype. This transition facilitates hematoma clearance, tissue repair, and anti-inflammatory responses through the release of cytokines such as IL-4 and IL-10. Furthermore, M2-polarized microglia promote the differentiation of Th2 and regulatory T cells, which further suppress M1 and Th1 phenotypes (18). As M2-type microglia differentiate, they contribute to BBB reconstruction and the reduction of cerebral edema, thereby alleviating mass effect. This process helps disrupt the cycle of escalating inflammation and fosters an environment conducive to brain tissue repair (80). As shown in **Figure 2**. Therefore, it is widely believed that facilitating the transition of microglia toward the M2 phenotype after intracerebral hemorrhage may alleviate brain injury and improve neurological outcomes in patients.

In recent years, there has been heightened focus on the functional polarization of microglia following a stroke, especially regarding the equilibrium between their pro-inflammatory and anti-inflammatory states. Studies have shown that activating signaling pathways such as Nrf2 (84), PPARγ/RAD21 (85), and TGF-β/ALK5 (86), or inhibiting inflammatory pathways such as TLR4/NF-κB and CDK5/DRP1 (87, 88), can promote microglial polarization toward the M2 phenotype, enhance phagocytic capacity, reduce neuroinflammation, and accelerate hematoma clearance and neural repair. In addition, miRNAs such as miRNA-182-5p and miRNA-27a suppress M1 microglial activation by targeting inflammatory mediators like TLR4 (89, 90). Meanwhile, miRNA-144 and miRNA-222 regulate pathways involving mTOR, autophagy, or apoptosis, thereby further modulating microglial inflammatory responses (91–93). These findings underscore the essential importance of regulating microglial functional states, particularly by inhibiting M1 activation and promoting M2 polarization, in the treatment and prognosis of stroke (94).

Although the “microglial polarization” and M1/M2 dichotomy are still widely used in the literature, studies have shown that this model oversimplifies the situation, particularly in *in vivo* environments (95, 96). In recent years, single-cell transcriptomics and spatial omics have revealed that microglia exhibit a continuous spectrum of subpopulations under injury and disease conditions, surpassing traditional classifications (97, 98). Among them, disease-associated microglia are characterized by TREM2–APOE signaling and lipid metabolism reprogramming, playing a role in phagocytosis and inflammation regulation (99). White matter-associated microglia and interferon-responsive microglia reflect specific responses to aging, demyelination, or viral-like stimuli (100, 101). However, there is limited evidence regarding the phenotypic characteristics of these microglial cells in the context of intracerebral hemorrhage, and the understanding of their dynamic changes and functional roles remains incomplete.

Microglia are crucial in the immune response after a stroke. Adjusting the equilibrium between pro-inflammatory and anti-inflammatory states can effectively alleviate brain injury and promote neural repair. Therefore, developing therapeutic strategies that target microglial function, particularly through the regulation of their activation states, may offer novel approaches for immunotherapy in stroke.

### 2.3 Neutrophils in intracerebral hemorrhage

Neutrophils, the predominant leukocytes in the peripheral immune system, are among the initial cells recruited to the central nervous system after intracerebral hemorrhage (33), where they are pivotal in the immune response (33, 102, 103). Studies have shown that the BBB becomes permeable as early as 3 minutes after brain injury (71). Neutrophil infiltration can be observed within 30 minutes and peaks at 2 to 3 days post-injury (104).

**Figure 3:**
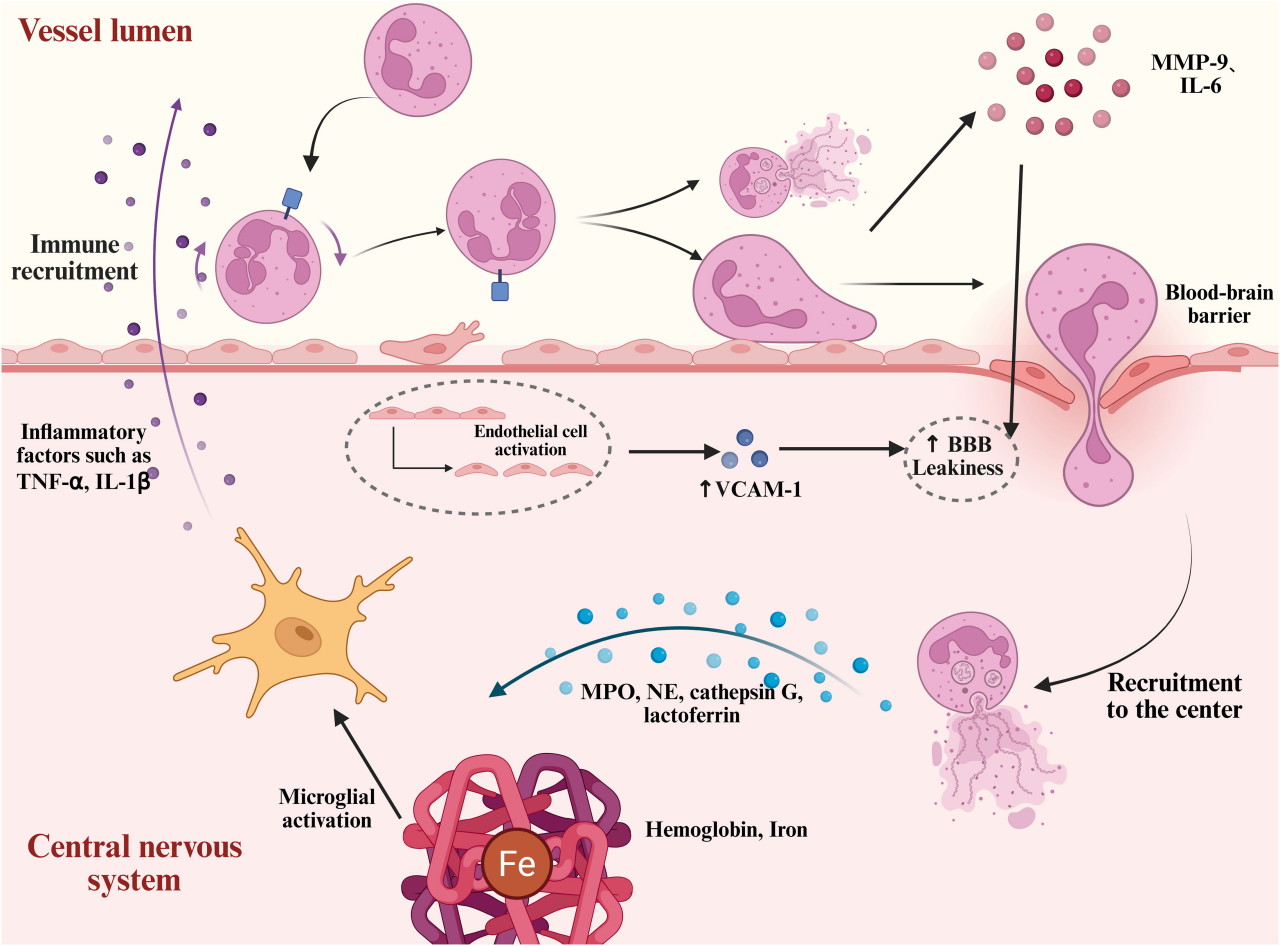
Neutrophil mobilization and neurotoxic mechanisms after intracerebral hemorrhage. After ICH, hemoglobin, iron, and other damage-associated molecules induce endothelial cell activation. This process is accompanied by the upregulation of adhesion molecules such as VCAM-1, which enhances neutrophil adhesion to the endothelium. Subsequently, neutrophils disrupt and traverse the BBB, migrating into the central nervous system. They release pro-inflammatory granules such as neutrophil elastase, cathepsin G, and lactoferrin. In addition, neutrophils form NETs, which, together with inflammatory mediators such as MMP-9 and IL-6, exacerbate BBB disruption and neuronal injury.

As shown in **Figure 3**, the marked increase in BBB permeability after brain injury facilitates the transendothelial migration of neutrophils. This process is primarily driven by DAMPs, which rapidly activate endothelial cells and enhance the expression of various cytokines, including vascular cell adhesion molecule-1, a key mediator of peripheral leukocyte adhesion and migration (105, 106). This activation can persist up to five days post-injury (107). Numerous studies have shown that targeting cell adhesion molecules, such as VLA-4, can reduce neutrophil adhesion to endothelial cells, significantly attenuating microglial activation and neuronal damage in the brain parenchyma (108). In addition, neutrophil-derived MMP-9 plays a critical role in this process. Numerous studies have demonstrated that, in subarachnoid hemorrhage models, neutrophil-released MMP-9 and IL-6 further disrupt the integrity of the BBB, promoting the infiltration of peripheral immune cells and perpetuating a vicious cycle of inflammation (109–112). Moreover, activated neutrophils not only exert direct cytotoxic effects on brain tissue, but also exacerbate local inflammation through interactions with other immune cells such as microglia and macrophages. Studies have shown that neutrophils can activate microglia, promoting the release of pro-inflammatory cytokines and amplifying the inflammatory response, ultimately worsening neuronal injury (113).

Neutrophils are considered a key component of the innate immune system’s initial response to microbial invasion (114). Neutrophil migration is not merely a simple chemotactic process; it also involves dynamic changes in their functional states. Before stimulation, naïve neutrophils exhibit a spherical morphology characterized by pronounced membrane ruffles. Upon stimulation with IL-8, phorbol myristate acetate, or lipopolysaccharide, they exhibit morphological alterations marked by a flattened morphology and the development of membrane protrusions (21). Following ICH, neutrophils transition from a resting to an activated state during their recruitment to the central nervous system. This activation involves the assembly of NADPH oxidase components (NOX), massive production of reactive oxygen species (ROS), cytoskeletal rearrangement, and initiation of the degranulation process (115). Neutrophil degranulation releases a variety of contents, including MPO, neutrophil elastase (NE), cathepsin G, and lactoferrin, which contribute to the clearance of necrotic tissue and pathogens. However, these granules also exhibit significant neurotoxicity. In particular, when combined with extracellular DNA to form NETs, they are considered key contributors to secondary neuronal injury (116). As shown in **Figure 3**.

## 3 NETs in intracerebral hemorrhage

### 3.1 Mechanisms of NETs formation and physiological functions

#### 3.1.1 Formation and regulation of NETs

**Figure 4:**
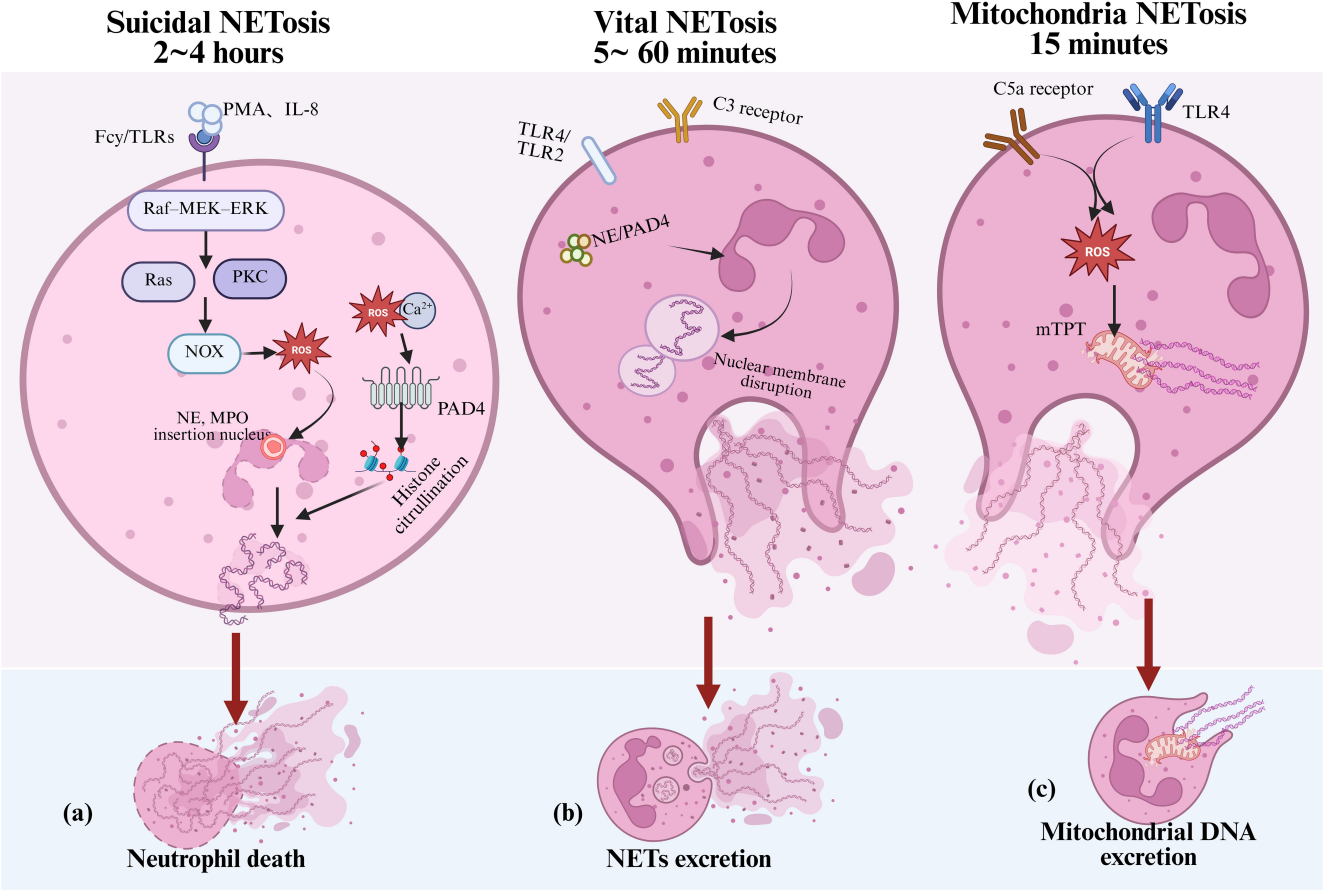
Mechanisms of NETs formation in inflammation. Under inflammatory stimulation, neutrophils can form NETs through three distinct mechanisms, contributing to both immune defense and tissue damage: **(a)** suicidal NETosis, which depends on NADPH oxidase activity and the accumulation of ROS; **(b)** vital NETosis, which is independent of NADPH oxidase and typically triggered by TLRs or complement component C3; and **(c)** mitochondrial NETosis, which is induced by factors such as LPS and C5a, leading to the release of mitochondrial DNA to form NETs. NETs, neutrophil extracellular traps; ROS, reactive oxygen species; NE, neutrophil elastase; MPO, myeloperoxidase; PAD4, Peptidylarginine deiminase 4; TLRs, toll-like receptors; LPS, lipopolysaccharide; mtDNA, mitochondrial DNA; mPTP, mitochondrial permeability transition pore; PKC, protein kinase C.

Takei was the first to observe that neutrophils can form NETs to kill bacteria (117). Brinkmann et al. further elucidated this process in 2004, during which they also introduced the term “NETosis.” (21) With continued research, it has become increasingly evident that the release of extracellular DNA is not invariably linked to cell death (22, 118, 119). In 2018, the Nomenclature Committee on Cell Death proposed substituting the term “NETosis” with “NETs” to more accurately describe this complex biological process (118). The formation of NETs is a highly regulated and programmed event, controlled by various endogenous and exogenous signals. Based on whether it is dependent on cell death or NOX activity, NETs formation can be categorized into three distinct mechanisms. As shown in **Figure 4**.

Suicidal NETosis is the earliest identified and most well-characterized form of NET formation. It depends on the activation of NOX and the production of ROS, typically occurring 2 to 4 hours after neutrophil activation. Specifically, stimuli such as phorbol myristate acetate and IL-8 activate Fcγ receptors or Toll-like receptors (TLRs), initiating the Raf–MEK–ERK signaling cascade. This sequentially activates Ras and protein kinase C (PKC), ultimately driving NOX activity and the production of ROS (120). The accumulation of ROS plays a central role in NETosis. On one hand, ROS promotes the translocation of NE and MPO into the nucleus, where they cooperatively mediate chromatin decondensation (121). On the other hand, extracellular calcium influx, together with ROS, activates peptidylarginine deiminase 4 (PAD4), which catalyzes the citrullination of histones H3, H4, and H2A. This modification weakens the electrostatic interactions between histones and DNA, leading to chromatin relaxation (122, 123). Ultimately, nuclear envelope rupture allows decondensed DNA to combine with granule proteins and form the characteristic NET structure, which is then released into the extracellular space. As shown in **Figure 4**, this process is typically accompanied by programmed neutrophil death.

However, studies have also shown that neutrophils can rapidly release NETs without undergoing cell death, a process termed “vital NETosis.” This form of NET release, as shown in **Figure 4**, is characterized by its independence from NADPH oxidase activity and is primarily induced by stimuli such as TLR2/TLR4 and complement component C3 (124). Vital NETosis typically transpires within 5 to 60 minutes after neutrophil activation and involves nuclear envelope vesiculation and the extrusion of DNA. During this process, neutrophils retain key immune functions such as phagocytosis and chemotaxis (125).

Further studies have identified a third type of NET formation termed “mitochondrial NETosis,” (126) which is induced by stimuli such as lipopolysaccharide, complement component C5a, and granulocyte-macrophage colony-stimulating factor (GM-CSF). As shown in **Figure 4**. This process typically occurs within 15 minutes after neutrophil activation. This process does not depend on nuclear DNA release. Instead, it is driven by the opening of the mitochondrial permeability transition pore (mPTP) and the generation of mitochondrial reactive oxygen species, which trigger the release of mitochondrial DNA and the formation of NETs without causing cell death (126). The three NET formation mechanisms occur at different time points following neutrophil activation, may or may not depend on NADPH oxidase activity, and can be associated with or independent of neutrophil death. However, the precise mechanisms underlying NET formation remain controversial and require further investigation.

#### 3.1.2 Physiological functions of NETs

**Figure 5:**
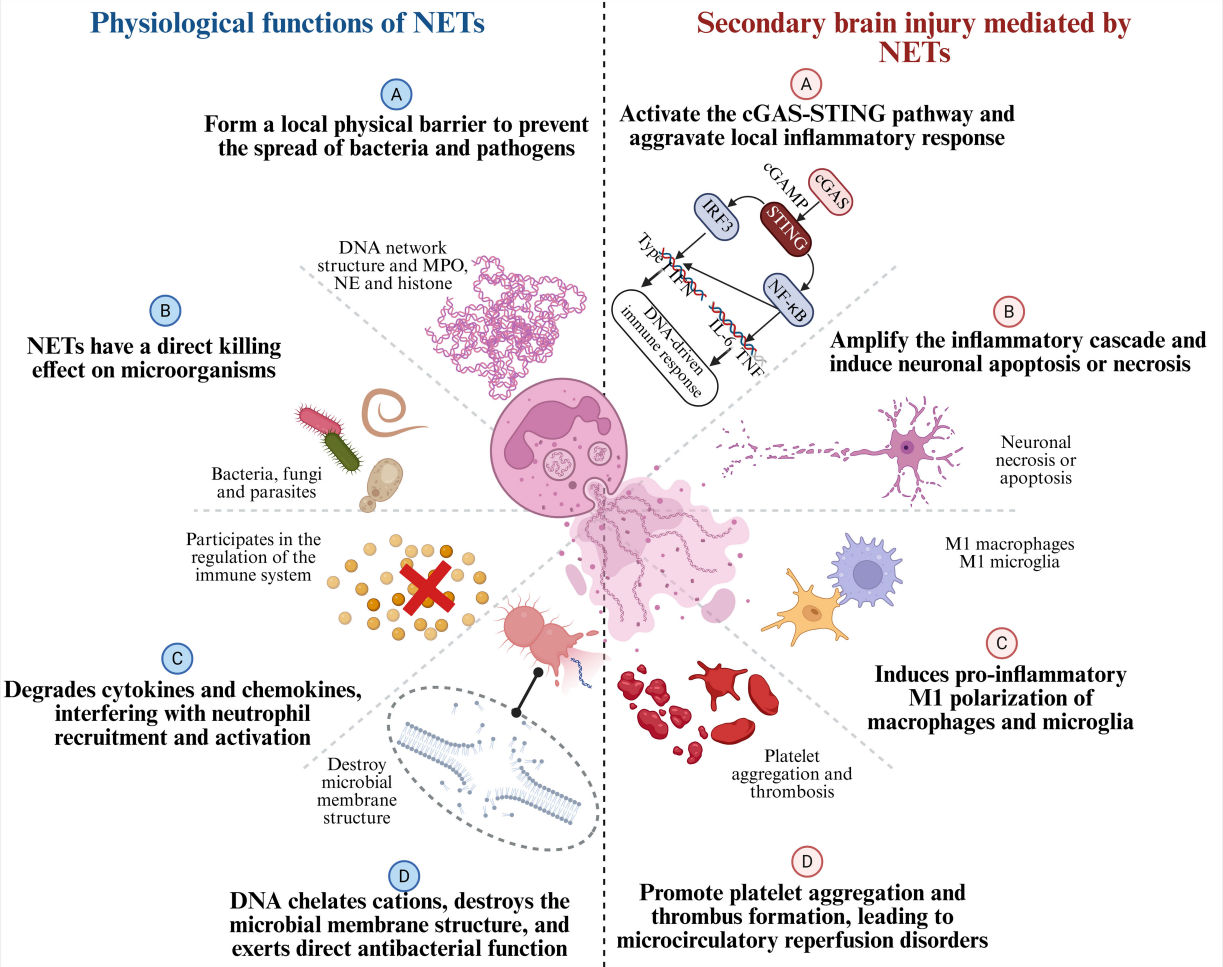
Physiological functions of NETs and their roles in secondary brain injury after ICH.NETs are composed of chromatin, histones, NE, and MPO. On the left, the physiological functions of NETs are shown: **(A)** forming a local physical barrier to prevent the spread of pathogens; **(B)** exerting direct killing effects on microorganisms; **(C)** degrading cytokines and chemokines to regulate neutrophil recruitment and activation; and **(D)** chelating cations, disrupting microbial membranes, and exerting direct antibacterial activity. On the right, after ICH, the detrimental effects of NETs as pro-inflammatory factors are shown: **(A)** activating the cGAS-STING pathway and aggravating local inflammation; **(B)** amplifying the inflammatory cascade and inducing neuronal apoptosis/necrosis; **(C)** promoting pro-inflammatory M1 polarization of macrophages and microglia; and **(D)** enhancing platelet aggregation and thrombosis, leading to microcirculatory disturbances.

As a major effector mechanism of neutrophils, NETs were initially thought to play a key role in host defense, particularly against bacterial and viral infections (127). The DNA web-like structures, together with antimicrobial components such as MPO, NE, and histones, form a local physical barrier that restricts the spread of bacteria and pathogens (21), as shown in **Figure 5**. Studies have confirmed that NETs exert direct microbicidal effects against a variety of pathogens, including bacteria, fungi, and parasites (128). However, accumulating evidence indicate that NETs are involved not only in pathogen defense but also in the modulation of various aspects of the immune response (129). Antimicrobial components within NETs, such as NE, can target and degrade various bacterial virulence factors (130), thereby reducing their pathogenicity (131). MPO contributes to bacterial killing through the release of ROS (132). In addition, the DNA backbone of NETs carries a natural negative charge, which enables it to chelate cations and disrupt microbial membrane integrity, thereby exerting direct antimicrobial effects (133). When exogenous DNase is used to degrade NETs in mice, the structural integrity of NETs is disrupted, leading to a significant increase in bacterial load. This further confirms the barrier function of NETs in innate immunity (134). In addition to their direct antimicrobial functions, NETs also regulate immune responses in sterile inflammatory environments. NETs aggregation can degrade cytokines and chemokines, thereby interfering with neutrophil recruitment and activation, and ultimately suppressing the inflammatory response (23). Moreover, NETs are believed to play a role in the onset of autoimmune responses under certain conditions, potentially through mechanisms such as antigen citrullination.

Notably, under the immunologically active conditions of stroke, NETs exhibit a complex dual role in physiological function (17). While NETs contribute to immune defense, their excessive formation can lead to increased immune cell recruitment, amplify the inflammatory response, and exacerbate post-stroke tissue damage through the release of various cytokines and enzymes. Therefore, precise regulation of NET formation may represent a promising therapeutic approach in stroke immunomodulation.

### 3.2 NETs-mediated secondary brain injury

In recent years, research on NETs has provided novel insights into the pathogenic mechanisms of neutrophils. NETs are web-like structures composed of decondensed chromatin, histones, MPO, NE, and other associated proteins. Their formation process is known as NETosis. Significant accumulation of NETs has been observed in both ICH models and clinical specimens. A postmortem study by Laurent Puy and colleagues was the first to demonstrate the presence of abundant NETs in the human brain following ICH (135). These NETs were predominantly localized within and around the hematoma, and their release exhibited temporal dynamics—typically initiating within 72 hours after hemorrhage and persisting over the following 8 to 15 days. Jin et al. reported the presence of NETs in patients with ICH (136). They detected abundant NETs in hematoma, plasma, and drainage fluid samples from ICH patients, suggesting that NETs play a significant role in the pathophysiology of hemorrhagic stroke.

The formation of NETs depends on the activation of multiple signaling pathways, including NOX2-mediated production of ROS and activation of TLR4 (137), both of which have been identified as critical triggers for NET generation. Moreover, following ICH, interactions between platelets and neutrophils have also been shown to promote the formation of NETs. The binding of P-selectin to P-selectin glycoprotein ligand-1 (PSGL-1) on neutrophils further promotes the formation of NETs (138). Recent studies have demonstrated that blocking PSGL-1 reduces plasma levels of MPO-DNA complexes (139), inhibits NET formation, and alleviates secondary brain injury. In addition, elevated platelet counts can enhance high mobility group box 1 (HMGB1)-mediated NET formation (140). Notably, heme, a key oxidative stress byproduct following ICH, has also been shown to promote the release of NETs by inducing ROS and activating NOX2 (141).

Although NETs formation plays an important physiological role in acute infection defense, as shown in **Figure 5**, NETs themselves also exert multiple direct pathogenic effects. Components of NETs, including NE, MPO, cathepsin G, and citrullinated histone H3 (CitH3), can amplify inflammatory cascades in the extracellular space, leading to neuronal apoptosis or necrosis. The DNA backbone within NETs acts as a potent immunostimulatory molecule that activates the cGAS-STING pathway, exacerbates local inflammatory responses, and induces pro-inflammatory M1 polarization of macrophages and microglia (102). Additionally, by promoting platelet aggregation and thrombosis, NETs may contribute to microcirculatory reperfusion impairment in the later stages of stroke, representing a potential barrier to neurological recovery. As we and others have previously demonstrated, several well-characterized signaling proteins have been demonstrated to modulate the formation of NETs, including JNK, extracellular signal-regulated kinases 1/2, Akt, and Src (142–145). Moreover, phorbol 12-myristate 13-acetate, a PKC activator, has been extensively utilized as a potent inducer of NETs in fundamental research (146–148). The participation of various signaling proteins suggests that the mechanism underlying NETs formation is highly complex. Notably, inhibiting the formation of NETs has emerged as a promising neuroprotective strategy following ICH. In animal studies, the use of PAD4 inhibitors or systemic administration of Deoxyribonuclease I (DNase I) effectively reduces NET levels, alleviates vascular inflammation, decreases hemorrhage volume, and significantly improves neurological recovery (149). More importantly, NETs may also serve as a potential biomarker for stroke prognosis, as elevated plasma NET levels in acute stroke patients have been reported to positively correlate with neurological deficit scores (150). With continued advances in understanding the mechanisms of NETs formation, therapeutic strategies targeting NETs hold promise as an innovative strategy for the management of ICH.

### 3.3 Mechanisms of NET-induced programmed cell death in ICH

**Figure 6:**
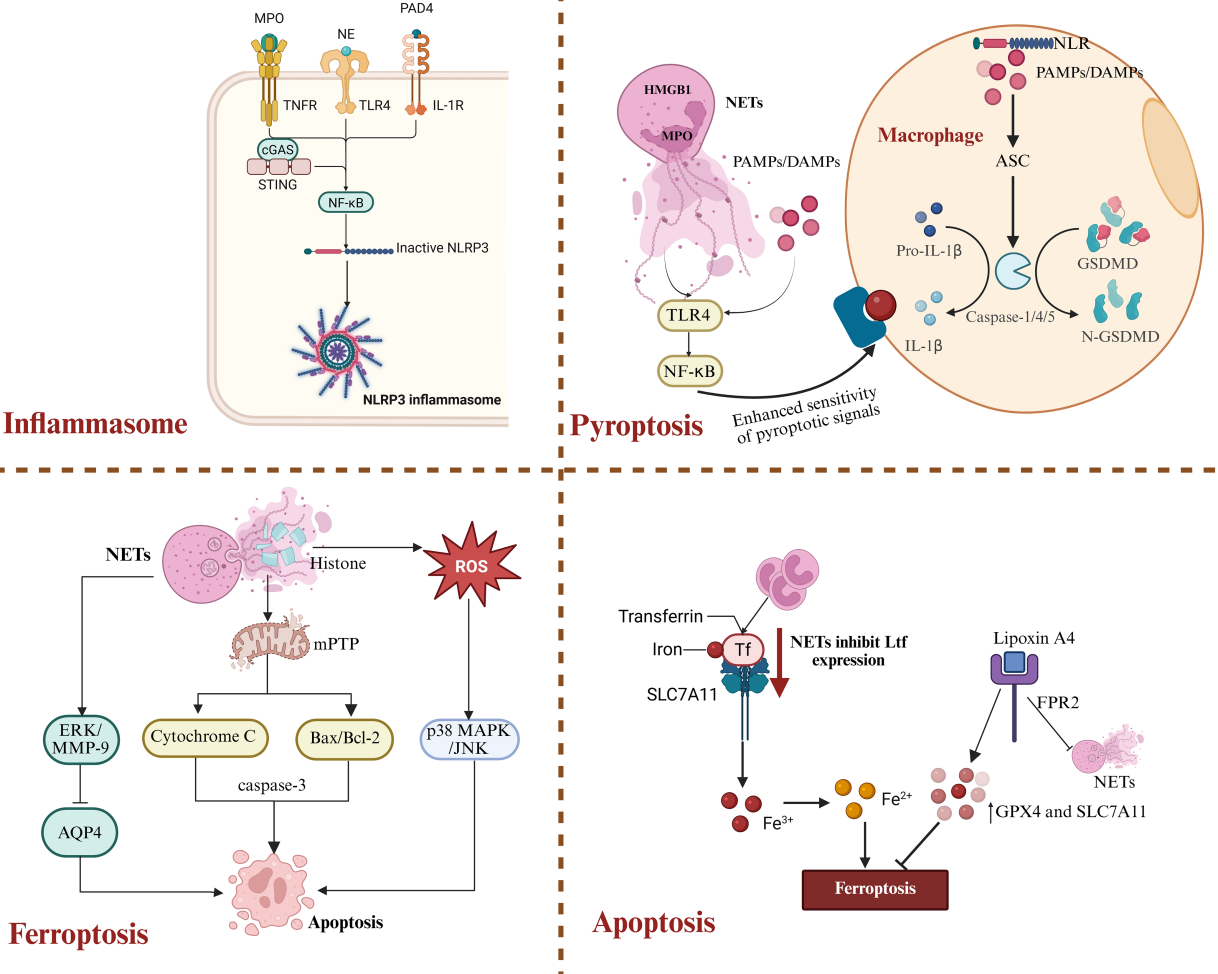
NETs-driven inflammatory cell death pathways after ICH. NETs play multiple roles in regulating neuronal cell death after ICH. By activating the NLRP3 inflammasome and various signaling pathways, NETs can induce pyroptosis, ferroptosis, and apoptosis, thereby exacerbating secondary brain injury.

Although NETs contribute to pathogen clearance during stroke, their “double-edged sword” effect in the immune response should not be overlooked. Aberrant activation and excessive formation of NETs, as shown in **Figure 6**, can trigger intense immune reactions and initiate multiple forms of programmed cell death—including pyroptosis, apoptosis, and ferroptosis—ultimately contributing to secondary neuronal injury (151).

Histones, MPO, NE, and PAD4, which are abundantly present in NETs, are considered classical DAMPs. These components can be recognized by mononuclear phagocyte lineages in the brain and activate the NLRP3 inflammasome pathway. Multiple studies have confirmed that the lesion area of ICH is often associated with high expression of caspase-1, IL-1β, and MPO (152, 153). In addition, extracellular DNA can activate the type I interferon pathway via the cGAS-STING axis, promoting pro-inflammatory polarization of microglia and exacerbating local neuroinflammatory responses (102). NETs induce activation of the TLR4/MyD88/NF-κB signaling axis through the specific binding of their histones to TLR4, thereby driving NF-κB dependent transcription of the IL-1β precursor (151). Moreover, the inflammasome contributes to atherosclerosis through this mechanism by inducing an interferon-α mediated neuroimmune cascade that amplifies immune cell recruitment within the plaque (154). This positive feedback loop involving the inflammasome, NETs, and TLR4 further impedes neural repair in the damaged area. As illustrated in **Figure 6**.

Pyroptosis is a highly inflammatory form of programmed cell death that primarily depends on caspase-1, -4, or -5-mediated cleavage of Gasdermin D, which leads to the formation of membrane pores and promotes the release of pro-inflammatory cytokines such as IL-1β. Upon sensing pathogen-associated molecular patterns (PAMPs) or DAMPs, NOD-like receptors (NLRs) recruit caspase-1 via the adaptor protein ASC, resulting in caspase-1 oligomerization and the induction of pyroptosis (155, 156). In addition, non-canonical inflammasome activation occurs when cytosolic lipopolysaccharide is sensed by caspase-4 (157, 158), leading to its activation, Gasdermin D pore formation, and subsequent activation of caspase-1 through the NLRP3 inflammasome. This mechanism has been confirmed in experiments using human-derived immune cells (159, 160). Components of NETs, such as MPO and high mobility group box 1, enhance macrophage sensitivity to pyroptotic signals and trigger their inflammatory, lytic cell death (154). Notably, in ICH models, cleavage products of Gasdermin D-N are frequently detected in the peritoneal region associated with NETs, indicating a prominent role of NETs in pyroptosis induction (161). Moreover, NETs-induced pyroptosis depends on the activation of the TLR4-NF-κB signaling axis (162). This process is not only mediated by NLRP3-driven caspase-1 activation but is also intricately linked to increased levels of ROS and activation of the HMGB1-TLR4 pathway (163, 164). DAMPs released during pyroptosis, in turn, promote the formation of NETs, establishing a self-amplifying “pyroptosis-NETs” inflammatory loop (165).

Ferroptosis, a form of programmed cell death characterized by iron dependency and the accumulation of lipid peroxides, has increasingly been recognized as a key contributor following ICH. Studies have shown that ICH-induced neutrophil infiltration reduces the transcription and expression of lactoferrin, thereby weakening its interaction with SLC7A11 and indirectly exacerbating neuronal ferroptosis (166). Animal model studies further demonstrate that Lipoxin A4 significantly inhibits the formation of NETs via an FPR2-dependent mechanism, thereby upregulating the expression of GPX4 and SLC7A11 and ultimately suppressing ferroptosis (167). Additionally, in fluoride-induced brain inflammation, NETs have been shown to disrupt calcium homeostasis, thereby inducing neutrophil self-death and promoting a feedback loop of NETs release (168). The interaction between NETs and ferroptosis has not yet been fully elucidated; however, their coupling under hypoxic conditions in the early stages of ICH has been demonstrated in multiple animal models, highlighting a promising emerging target for further investigation.

In addition to pyroptosis and ferroptosis, NETs can also induce neuronal apoptosis. Extracellular histones released from NETs directly disrupt the integrity of the cell membrane, trigger the opening of mitochondrial permeability transition pores, lead to cytochrome c release and Bax/Bcl-2 imbalance, and ultimately activate the caspase-3-dependent apoptotic pathway (169). Moreover, NETs-induced ROS stress activates the p38 MAPK and JNK signaling pathways, further amplifying cell death signals (170). However, the role of NETs in inducing apoptosis following ICH has not yet been systematically elucidated. Recent studies have shown that NETs disrupt the BBB by activating the ERK/MMP-9 signaling pathway and suppressing the expression of AQP4, leading to perihematomal edema and neuronal apoptosis. This represents one of the key mechanisms underlying secondary brain injury after ICH. In summary, NETs aggravate secondary injury to the central nervous system by triggering multiple forms of programmed cell death.

## 4 Regulatory role of microglia in ICH

Following ICH, microglia, the resident immune cells of the central nervous system, are among the first to be activated. Their functional and phenotypic changes perform a pivotal function in both secondary neuronal injury and tissue repair. Through interactions with astrocytes, oligodendrocytes, and peripheral immune cells, microglia help establish a complex immunoregulatory network that exerts both detrimental and reparative effects on tissue injury and recovery after ICH.

### 4.1 Dual roles of microglia in the CNS immune network

**Figure 7:**
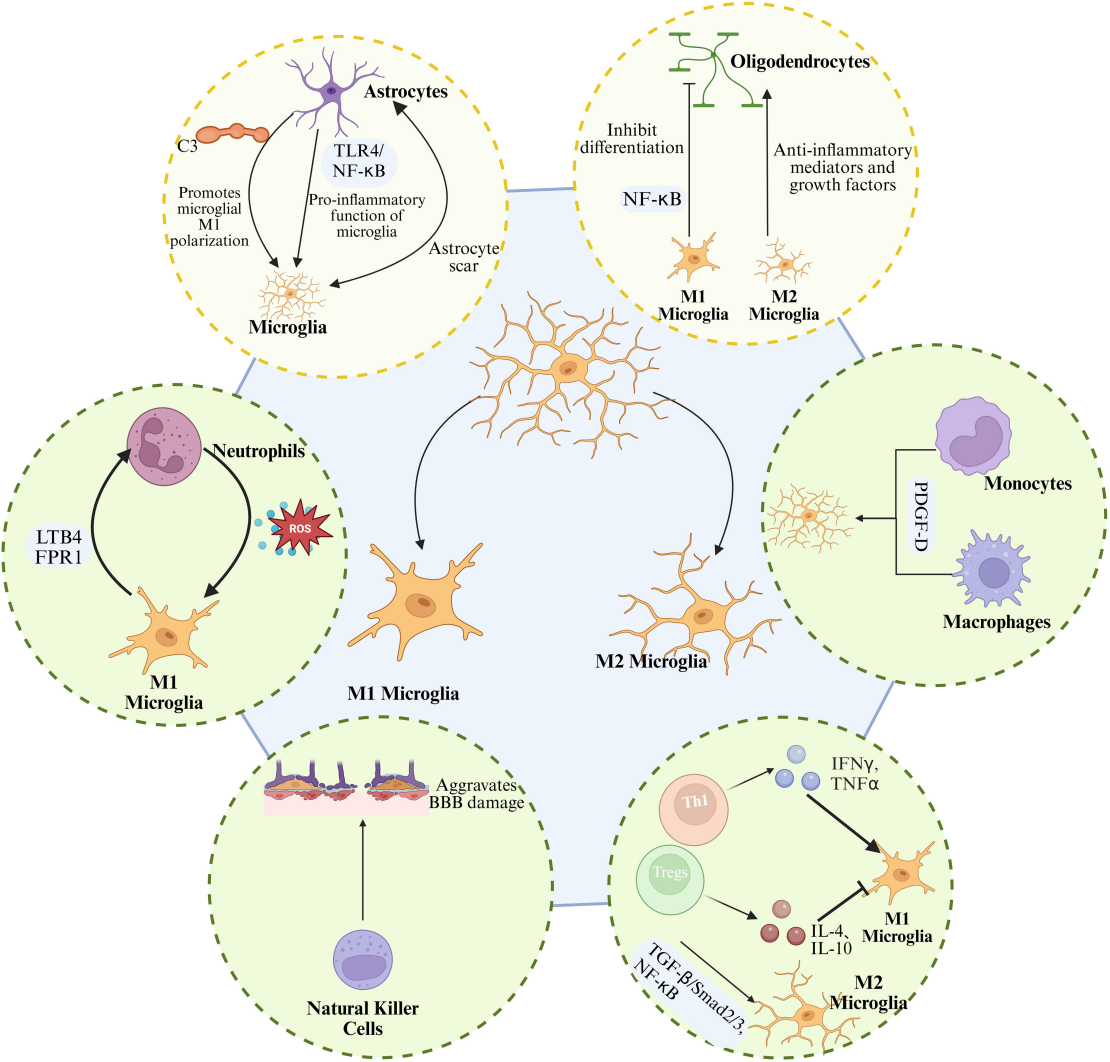
Microglial interactions with CNS and peripheral immune cells after ICH. In the CNS, microglia interact with astrocytes through multiple signaling pathways to regulate inflammation, glial scar formation, and neural repair. Moreover, microglial polarization influences oligodendrocyte differentiation and myelin regeneration. In the periphery, microglia release chemokines to interact with neutrophils, macrophages, natural killer (NK) cells, and T lymphocytes, establishing a complex network of inflammatory amplification and immune regulation.

In the early stages of ICH, components such as hemoglobin and heme rapidly activate microglia via the TLR4, driving their polarization toward the pro-inflammatory M1 phenotype and resulting in the release of large amounts of DAMPs (81, 82). Molecules such as HMGB1, heat shock proteins, and nucleic acid fragments can bind to pattern recognition receptors (PRRs) on the surface of microglia, such as TLR4 and TLR2. This interaction triggers intracellular signaling via the MyD88 and TRIF pathways, leading to the activation of transcription factors such as NF-κB and the subsequent initiation of pro-inflammatory gene expression (171, 172). Moreover, microglial recognition of PRRs such as NLRP3 further amplifies the inflammatory cascade, thereby contributing to a pro-inflammatory microenvironment (171). Additionally, thrombin enhances the pro-inflammatory activation of microglia through protease-activated receptor-1. This process increases BBB permeability, facilitates the infiltration of inflammatory cells and harmful molecules into brain tissue, and exacerbates cerebral edema and neuronal injury. However, this inflammatory response may also confer short-term protective effects by aiding in the clearance of necrotic tissue and limiting lesion expansion. As shown in **Figure 7**.

Over time, activated microglia progressively shift toward the M2 phenotype, releasing anti-inflammatory cytokines such as IL-10 and TGF-β, as well as neurotrophic factors. This phenotypic transition contributes to hematoma clearance, vascular regeneration, and axonal repair, thereby reducing inflammation and improving neurological outcomes. This phenotypic shift is not only influenced by changes in the inflammatory microenvironment but also regulated by signaling pathways such as JAK1/STAT6, Nrf2, and PPARγ. For example, activation of Nrf2 enhances the phagocytic capacity of microglia and increases the expression of neurotrophic factors (84). In addition, upregulation of the PPARγ (85) and TGF-β/ALK-5 pathways (86), or suppression of key inflammatory mediators such as the TLR4/NF-κB pathway (87) and the CDK5)DRP1 axis (88), contributes to M2 polarization and inhibition of pro-inflammatory responses. Moreover, the regulatory role of microRNAs is also significant (173). For instance, miR-144 targets the mTOR pathway to modulate autophagic activity in microglia, contributing to hemoglobin-induced inflammatory responses (91–93). Meanwhile, miR-27a and miR-182-5p attenuate the release of pro-inflammatory cytokines by inhibiting downstream signaling of the TLR4 pathway (89, 90).

Within the central immune network, interactions between microglia and astrocytes are particularly intimate. A study by Shi et al. demonstrated that IL-15 exacerbates cerebral edema and neuronal injury after ICH by enhancing inflammatory signaling between astrocytes and microglia (174). In addition, the complement system serves as a critical communication pathway between astrocytes and microglia. A1-type astrocytes secrete complement component C3, which activates microglia through the C3/C3aR axis, promoting M1 polarization and exacerbating inflammation and white matter injury (175, 176). Moreover, overexpression of aquaporin-2 in astrocytes can induce inflammatory cascades by activating the TLR4/NF-κB signaling pathway, thereby enhancing the pro-inflammatory activity of microglia (177). Notably, microglia play roles not only in regulating inflammation but also in coordinating with astrocytes to modulate glial scar formation, thereby influencing brain tissue repair (178). Research indicated that astrocytes secrete functional mitochondria and neurotrophic factors, including brain-derived neurotrophic factor, which can be transferred to microglia. This transfer promotes microglial polarization toward the M2 phenotype, enhances their phagocytic and antioxidant capacity, reduces neurotoxicity, and facilitates the remodeling of neural networks (179).

White matter injury is a common consequence of ICH, characterized by oligodendrocyte death and demyelination. Microglia also play a critical role in both the injury and repair of white matter (175). Under the oxidative stress induced by ICH, M1-polarized microglia inhibit the differentiation of oligodendrocyte precursor cells into mature oligodendrocytes via NF-κB signaling, thereby impairing remyelination. In contrast, M2 microglia promote OPC maturation and remyelination by releasing anti-inflammatory mediators and growth factors. Therapeutic strategies targeting this pathological process, such as the application of nanoceria, have been shown to alleviate myelin damage and enhance remyelination.

### 4.2 Crosstalk and reciprocal regulation between microglia and peripheral immune cells

In the context of peripheral immune responses, microglia promote the infiltration of peripheral immune cells into brain tissue by releasing chemokines and cytokines, with neutrophils playing a particularly prominent role in secondary brain injury, as shown in **Figure 7**. Neutrophils are actively recruited in response to microglia-derived leukotriene and signals mediated by formyl peptide receptor 1. Upon infiltration, they release ROS and pro-inflammatory cytokines, which in turn further activate microglia and exacerbate tissue damage (180, 181). Meanwhile, the infiltration of macrophages and monocytes enhances microglial activation (182–184) and modulates their pro-inflammatory responses through pathways such as platelet-derived growth factor D (185). Natural killer (NK) cells and T lymphocytes also participate in the functional regulation of microglia. NK cells exacerbate BBB disruption by damaging endothelial cells and inducing pro-inflammatory activation of microglia (186). T cells, particularly Th1 cells, promote M1 polarization by secreting IFN-γ (187), whereas regulatory T cells and Th2 cells suppress the M1 phenotype and enhance M2 functions by releasing anti-inflammatory cytokines such as IL-4 and IL-10 (188). After ICH, an increase in regulatory T cells is positively correlated with microglial M2 polarization. Tregs limit inflammatory damage and promote hematoma clearance through the TGF-β/Smad2/3 signaling pathway and by inhibiting NF-κB activation (79). Importantly, peripheral immune cells also regulate the M1/M2 polarization of microglia by releasing various inflammatory mediators. IL-4 and IL-10 promote the shift from the M1 to M2 phenotype through the JAK1/STAT6 signaling pathway (189).

In summary, microglia play a central role in regulating central immune responses after ICH through dynamic phenotypic transitions. They not only orchestrate the early inflammatory response but also engage in complex interactions with peripheral immune cells to shape both the pathological progression and repair outcomes. Elucidating the mechanisms by which microglia operate within this complex network, particularly their functional crosstalk with neutrophils, will provide a theoretical basis for developing novel immunotherapeutic strategies and may improve neurological outcomes following ICH.

## 5 Bidirectional regulation between NETs and microglia: from stroke to neurodegenerative diseases

Immune responses triggered by neurological disorders involve not a single immune cell population, but rather a complex network of cellular interactions. Within this network, the bidirectional regulation between NETs and microglia plays a critical role in shaping post-stroke immune responses. The interaction between NETs and microglia not only regulates local immune responses during the acute phase, but may also profoundly influence neurorepair, BBB integrity, and functional recovery in stroke and other neurological disorders.

After ICH, microglial phenotypes and functions exhibit significant temporal changes: during the acute phase (within approximately 72 hours), a pro-inflammatory or interferon-responsive phenotype predominates, gradually transitioning to a phenotype characterized by phagocytosis and repair, such as DAM-like (18, 32, 97, 190). Correspondingly, NETs are closely associated with the acute phase of ICH and disease progression, promoting perihematomal edema and vascular permeability disruption via the ERK-MMP9/AQP4 axis. Clearance or inhibition of NETs has been shown to reduce vascular and brain tissue damage (66, 191). In lineage studies, disease-associated microglia have been found to exhibit both anti-inflammatory or phagocytic and pro-inflammatory characteristics (192). Their activation depends on the triggering receptor TREM2 on the microglial surface, which facilitates phagocytosis of apoptotic neurons and induces the generation of a small number of pro-inflammatory factors (193). In animal models of ICH, TREM2 is activated around the hematoma after intracerebral hemorrhage, reducing neuroinflammation and inhibiting neuronal apoptosis (194). However, in-depth studies on the DAM concept have largely focused on neurodegenerative diseases like Alzheimer’s, and related evidence in ICH remains limited. Existing studies suggest that microglia with different phenotypes may influence the generation and clearance of NETs through distinct mechanisms at different stages of ICH. During the acute phase, pro-inflammatory/IFN-responsive phenotypes are more likely to synergize with neutrophil activation and NET formation, contributing to lesion clearance but potentially exacerbating secondary damage. In contrast, in the subacute to chronic phase, microglia that shift toward repair or DAM-like phenotypes may limit the persistence of NETs by enhancing phagocytosis and anti-inflammatory signaling, thereby promoting tissue remodeling and functional recovery (192). Although the proposed dichotomy between M1 and M2 phenotypes is considered overly simplistic, this classification remains important in studying microglial function.

However, current research on the interactions between NETs and microglia in ICH remains limited. Most available evidence is derived from models of ischemic stroke, TBI, and neurodegenerative disorders such as Alzheimer’s disease (AD). Despite variations in disease contexts, current studies consistently reveal a highly conserved crosstalk mechanism between NETs and microglia in central inflammatory responses. Therefore, systematically elucidating their synergistic interactions, particularly in the regulation of inflammation, BBB disruption, and neurological dysfunction, will help uncover the key pathological mechanisms underlying secondary neural damage following ICH. As presented in **Table 1**.

**Table 1:** Mechanisms of NET–microglia interaction.

Functional direction	Mechanistic effect	Molecular mechanism	Ref.
Regulation of Secondary Inflammation	Microglia recruit neutrophils and induce NETs release.	cGAS-STING	(102, 195)
	NETs induce microglial polarization and pro-inflammatory cytokine release.	cGAS-STING, C3aR, TREM1–SYK, HMGB1/JNK/AP1	(196–199)
BBB Modulation	Histones from NETs bind to endothelial cells and disrupt the cytoskeleton.	Histone–receptor binding	(200, 201)
	Degradation of extracellular matrix	ERK/MMP9/AQP4	(202, 203)
	Regulation of tight junction proteins and vascular remodeling	PAD4-STING-IFNβ pathway	(204)
Neurological and Cognitive Regulation	NETs-associated cytotoxins induce neuronal apoptosis	NETs-associated components	(205)
	Secondary inflammation exacerbates neuronal injury and cognitive impairment	M1 polarization and NETs interaction	(206)
	NETs accumulate in Aβ-rich regions, activate inflammation, and impair synaptic networks	NETs-Aβ interaction	(207, 208)

### 5.1 Coordinated regulation of the inflammatory response

Inflammation is a principal pathological characteristic of ICH, with secondary inflammatory responses significantly influencing stroke progression, the degree of neuronal damage, and subsequent neurological recovery. Microglia promote the release of NETs, which in turn further activate microglia. Together, they facilitate the recruitment of local immune cells and influence the pathological progression of the disease. As illustrated in **Table 1**. NETs and microglia, as core participants in the immune response, form a self-amplifying inflammatory loop through bidirectional regulation. Microglia promote neutrophil recruitment and NET release, while NETs, in turn, activate microglia, driving pro-inflammatory responses and further infiltration of immune cells, thus establishing a self-sustaining positive feedback loop of inflammation (209).

Existing direct evidence in ICH shows that this bidirectional interaction is rapidly initiated during the acute phase. Hematoma degradation products, DAMPs, and endothelial signals exposed by blood-brain barrier disruption (e.g., ICAM-1, P-selectin) can drive extensive neutrophil infiltration into the brain parenchyma (82, 102, 210). After entering the brain parenchyma, neutrophils form NETs in the local inflammatory environment, releasing extracellular DNA, histones, and proteases. These molecules directly interact with microglial pattern recognition receptors, inducing the release of pro-inflammatory factors and chemokines such as IL-1β, IL-6, MCP-1, and CXCL-1 (200), which further enhance neutrophil recruitment and NET formation (210). Pharmacological studies also support this shared regulatory pattern. Degradation of NETs (e.g., DNase I, PAD4 inhibitor GSK484) can reduce the proportion of pro-inflammatory microglia and the levels of inflammatory factors (210). In contrast, inhibiting microglial activation (e.g., C3aR antagonist, TREM1 inhibitor LP17, Mincle–Syk blocker albumin) not only reduces local inflammatory responses but also decreases neutrophil recruitment and NET formation (4, 7, 211).

**Figure 8:**
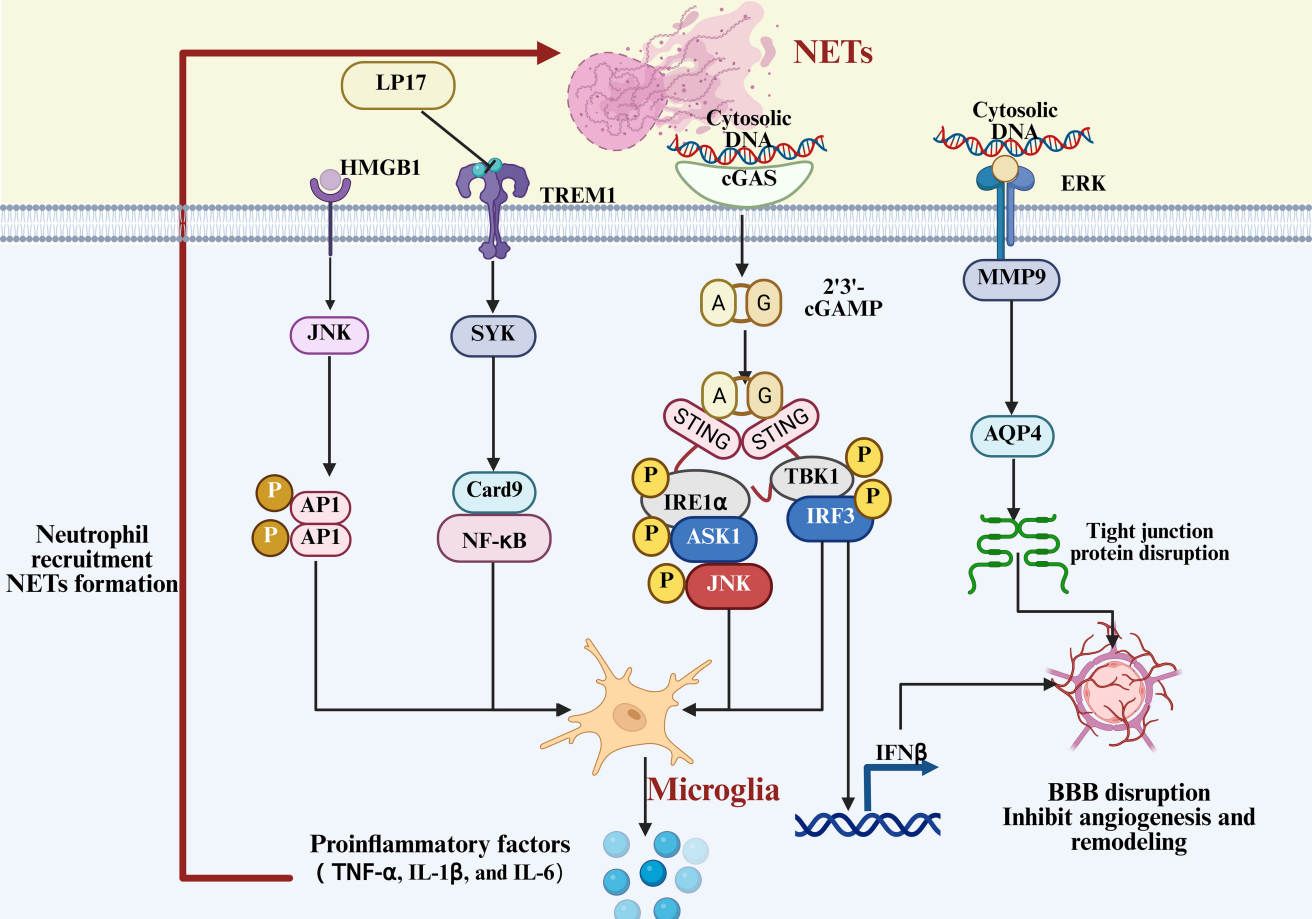
Mechanism of NET-induced microglial activation and BBB disruption. Neutrophil extracellular traps (NETs) activate microglia through multiple signaling cascades, amplifying neuroinflammatory responses and exacerbating blood-brain barrier disruption.

Research on this mechanism is continuously advancing, as shown in **Figure 8**. Studies have found that NETs can drive pro-inflammatory polarization and inflammation amplification in microglia through multiple signaling pathways. NETs contribute to neuroinflammation and BBB damage in tPA-related intracerebral hemorrhage by promoting the activation of the cGAS-STING pathway in microglia (102). In ICH, C3aR-mediated microglial activation promotes the expression of the NLRP3 inflammasome through the activation of the PKC/p38MAPK cascade. It also facilitates the infiltration of neutrophils into the perihematomal region, forming inflammation cell clusters marked by MPO, which further disrupt the blood-brain barrier and exacerbate cerebral edema (212). Additionally, in SAH, the activation of the TREM1–SYK axis drives microglial polarization toward a pro-inflammatory phenotype and activates the PAD4–NETs pathway, promoting neutrophil formation of NETs. HMGB1 released from NETs can exacerbate the inflammatory cascade through the JNK/AP1 pathway (201). This bidirectional regulation of central and peripheral immunity intensifies neuroinflammatory responses and secondary brain damage following SAH (136, 213).

Interestingly, the bidirectional interaction between the two is also prominent in studies of other neurological disorders. Studies have found that ginsenoside Rg1-loaded vesicles enhance microglial clearance of neutrophils, significantly reducing NET release and promoting neovascularization and brain tissue repair (214). Further studies have found that the intelligent nanomaterial system C-Lipo/CA, containing a PAD4 inhibitor, can suppress NETosis and the cGAS-STING pathway, alleviating neuroinflammation and modulating the inflammatory phenotype of microglia (195). Further studies in TBI have found that NETs regulate microglial pro-inflammatory responses through the STING-dependent IRE1α/ASK1/JNK signaling pathway (215). In addition to stroke and TBI, Zenaro et al. were the first to report the existence of neutrophil-microglia crosstalk and NETs in both the vasculature and parenchyma in AD (216). Although these findings originate from non-ICH models, the revealed inflammation amplification pattern provides insights into the potential mechanisms in ICH.

In conclusion, although substantial evidence supports the bidirectional regulation between NETs and microglia in various neurological disorders, relevant studies in ICH remain scarce, particularly clinical research. ICH has a unique pathological context, including hematoma-driven inflammation and iron-mediated toxicity, and its immune regulatory mechanisms may differ significantly from those in ischemic stroke and TBI. Future studies should integrate ICH-specific animal models and clinical samples to further clarify the true role of NETs and microglia interactions in the progression of ICH and their therapeutic potential. Additionally, this inflammation amplification not only affects the release of cytokines but also further disrupts the structure and function of the blood-brain barrier, becoming a key mechanism in post-stroke secondary injury.

### 5.2 Synergistic disruption of the BBB

The BBB is a multilayered structure consisting of brain microvascular endothelial cells, the basement membrane, astrocytic end feet, and tight junction proteins. It serves as a critical defense barrier of the CNS, protecting against the infiltration of exogenous toxins and immune cells. After ICH, the structural integrity of the BBB is rapidly compromised during the acute phase, allowing harmful molecules and peripheral immune cells to enter the brain parenchyma, exacerbating secondary neural damage. Among them, the bidirectional regulation between NETs and microglia is one of the key mechanisms underlying BBB damage. NETs and microglia synergistically contribute to a compound injury pathway affecting the structure and function of the BBB, as shown in **Table 1**, through mutual amplification of inflammatory mediators, intercellular chemotaxis, and cross-activation of signaling pathways (217). Following stroke, microglia are rapidly activated into a pro-inflammatory M1 phenotype, releasing cytokines such as IL-1β and IL-6 that directly disrupt tight junctions of endothelial cells (218). In parallel, they promote astrocyte activation and glial scar formation, indirectly impairing the reconstruction and functional recovery of the BBB (219, 220). In addition, chemokines such as CXCL1 released by microglia facilitate the recruitment of neutrophils into the central nervous system. While modulating NETs formation, they also indirectly affect the integrity of the BBB via NETs, as shown in **Figure 8**.

Research has demonstrated that following ICH, excessive formation of NETs is not merely a reactive product of immune cells and pathological signals, but also contributes to BBB disruption through multiple mechanisms upon recruitment to the central nervous system. Histones released by NETs bind to receptors on the surface of endothelial cells, increasing membrane permeability and disrupting the cytoskeleton, thereby impairing endothelial barrier function (196). Additionally, elastase and proteases such as MMP-9 in NETs can degrade the extracellular matrix and tight junction proteins, downregulating AQP4, thereby further loosening the BBB structure (66, 197). At the same time, NETs can form dense complexes with fibrinogen and plasminogen, obstructing fibrinolysis and indirectly prolonging hematoma compression and mechanical damage to the BBB (136, 198). Moreover, under hypoxic conditions, the adhesion between NETs and endothelial cells is enhanced, further exacerbating endothelial injury and increasing vascular permeability (174, 199). In contrast, inhibition of key NETs-forming enzymes such as PAD4 reduces post-stroke BBB breakdown and enhances vascular plasticity.

It is important to note that the role of NETs and microglia in BBB damage is not a unidirectional process. NETs induce microglial inflammatory polarization through the TLR4/RAGE pathway. In turn, microglia stimulate the release of NETs via signaling axes such as the C3aR complement system (212), TREM1–SYK (201), and Mincle–Syk (201), amplifying the inflammatory response and endothelial barrier damage. After BBB damage, it also reciprocally promotes the infiltration of peripheral immune cells, allowing the positive feedback loop between NETs and microglia to continue. In therapeutic exploration, several drugs have shown potential in animal models to simultaneously protect the BBB and regulate NETs–microglia interactions. For example, Adjudin reduces neutrophil infiltration and MMP-9 levels while promoting the polarization of microglia to an anti-inflammatory phenotype (221). Methylene blue, on the other hand, upregulates IL-10 through the Akt/GSK-3β/MEF2D signaling pathway, inhibiting peripheral immune cell infiltration and microglial activation (211). Therefore, protecting the BBB should be considered one of the core downstream targets for targeting NETs–microglia interactions.

However, direct evidence for ICH remains very limited, despite a substantial body of research in ischemic stroke and neurodegenerative diseases. In ischemic stroke, NETs regulate the PAD4-STING-IFNβ pathway, promoting BBB damage and inhibiting neovascularization and vascular remodeling (207). Edaravone, in contrast, upregulates tight junction protein expression by reducing NET formation (222). Furthermore, NETs have been shown to drive synergistic damage to both the intestinal and blood-brain barriers through the DDIT4/IL-1β pathway, leading to the development of colitis and AD (223).

In the immune response, microglia act not only as initiators of inflammatory amplification but also as potential stabilizers that sustain barrier disruption. NETs and microglia engage in bidirectional inflammatory crosstalk, creating a sustained pro-inflammatory positive feedback loop that not only exacerbates local inflammation but also directly accelerates the deterioration of BBB structure and function. This synergistic mechanism has been reported in ischemic stroke, TBI, and various neurodegenerative diseases, but direct evidence in ICH remains limited and mostly confined to animal models. Given the unique pathological context of ICH, such as hematoma-driven inflammation and iron-mediated neurotoxicity, these extrapolated mechanisms may not fully reflect the actual disease progression. Future research must systematically verify the specific role of NETs–microglia interactions in BBB damage and their potential for intervention, using ICH-specific models and clinical samples.

### 5.3 Regulation of neural injury and cognition

NETs and microglia may play a crucial role in neuroinjury and cognitive dysfunction following ICH. Cognitive dysfunction is a key factor in long-term disability and decreased quality of life in ICH survivors, affecting various aspects such as attention, executive function, memory, and information processing speed. In recent years, immune-inflammatory responses, particularly the interaction between NETs and microglia, have been considered one of the key mechanisms driving cognitive decline following ICH. Histones and proteases released from NETs can directly damage neurons, inducing apoptosis and necrosis, and initiating an inflammatory cascade that further exacerbates neuronal loss. Moreover, NETs can further induce M1 polarization of microglia, forming a positive feedback loop between microglia and NETs. Thus, the interaction between NETs and microglia, as shown in **Table 1**, contributes to neuronal injury and cognitive impairment in central nervous system disorders through multiple mechanisms, exacerbating central nervous system inflammation and neuronal loss.

Existing animal studies on ICH show that NETs-mediated microglial pro-inflammatory activation is closely associated with early brain injury and poor functional outcomes (25). Acerboside D modulates neutrophil activity and NET formation, reducing inflammation and improving neurological function, motor coordination, and learning and memory abilities. The mechanism may involve upregulation of NTSR1 expression, activation of the cAMP/PKAc signaling pathway, and inhibition of PAD4 and citH3 activity, thereby suppressing NET formation (224). Additionally, in the ICH experimental model, neutrophil depletion improves brain perfusion and alleviates neurological deficits and long-term prognosis (113). Although these results suggest that the NETs–microglia axis may be involved in cognitive impairment following ICH, direct and systematic studies validating the relationship between their interaction and cognitive function remain extremely limited.

Due to the limited direct evidence linking ICH to cognitive impairment, some studies have drawn insights from findings in other neurological disorders. In ischemic stroke models, NETs have been found to contribute to delayed neuronal injury (108). The main mechanisms include disrupting the BBB (225), promoting thrombosis, and acting as an activation platform for inflammasomes (226), which synergistically amplify the inflammatory response, thereby exacerbating neurological dysfunction (203). Notably, the interaction between neutrophils and microglia is not limited to stroke models. In patients with cerebral small vessel disease, plasma NETs biomarkers are elevated and negatively correlate with overall cognition, executive function, and information processing speed (227). A similar phenomenon has also been observed in an AD mouse model (216, 228, 229). Although these studies provide clues for inferring the potential mechanisms of ICH, the pathological characteristics of ICH differ significantly from those of ischemic or degenerative diseases.

With growing research interest, the interaction between microglia and neutrophils has emerged as a focal point. Neumann et al. were the pioneers in presenting *in vivo* evidence of physical interaction between microglia and infiltrating neutrophils (108, 230). Emerging intervention strategies in neuronal injury and cognition, such as the gut-brain axis and nanozyme therapy, have become widely studied in various neurological disease models (202, 231, 232). However, their effectiveness in ICH and related cognitive impairment has yet to be directly validated. However, in a high-intensity inflammatory environment, microglia not only struggle to clear the large amounts of NETs formed but may also be activated by them, transitioning to a pro-inflammatory state, further promoting the recruitment of peripheral immune cells and exacerbating neuronal injury and cognitive dysfunction (136, 195, 201).

Although studies in ICH models have shown the role of NETs and microglia in neuroinflammation and functional impairment, direct evidence linking them to cognitive function remains lacking. In contrast, diseases like ischemic stroke and AD have provided insights into the underlying mechanisms. However, ICH has a unique pathological context, and extensive *in vitro* and *in vivo* research is needed to further clarify the interactions between NETs and microglia after ICH and evaluate their feasibility as intervention targets.

## 6 Therapeutic strategies targeting NETs After ICH

Aberrant formation of NETs plays a critical role in secondary inflammation after ICH by amplifying inflammatory signaling, disrupting the BBB, and inducing neuronal cell death. NETs formation is closely associated with PAD4 mediated histone citrullination and ROS production mediated by NADPH oxidase. In addition, NETs are primarily composed of a DNA backbone, histones, MPO, NE, and other cytotoxic proteins and enzymes. Therefore, inhibiting NET formation and promoting NET clearance represent key therapeutic strategies targeting NETs in the context of ICH, as summarized in **Table 2**.

**Table 2:** Therapeutic strategies targeting NETs after ICH.

Regulatory approach	Therapeutic strategy	Mechanism of action	Drug	Ref.
Inhibition of NETs Formation	PAD4 Inhibition	Inhibits PAD4 activity, reduces histone citrullination, and prevents NET release.	Cl-amidine, F-amidine, GSK484	(233, 234)
	NOX/ROS Inhibition	Inhibits ROS production via NOX suppression, thereby reducing NET formation and oxidative stress.	Vitamin C, Curcumin, DPI	(208, 235, 236)
Promotion of NET Clearance	NE and MPO Inhibition	Inhibits NE and MPO activity, reducing NETs formation and inflammatory responses.	Isorhamnetin, Folic Acid, Thiocyanate	(237, 238)
	Histone Neutralization	Reduces NET formation and neuronal death by blocking histone-DNA complexes.	Monoclonal antibodies, FSAP	(169, 239)
	DNA Degradation	Degrades NETs by hydrolyzing extracellular DNA and nuclear proteins.	DNase I, Recombinant DNase I	(240)
Other Approaches	Actin Modulation	Modulates actin cytoskeletal remodeling to reduce NET release.	MICAL-1 inhibitors	(241)
	FH	Regulates C3b degradation to inhibit NET release and reduce local inflammation.	Soluble Factor H, Immobilized FH	(242)
	akebia saponin D	Inhibits PAD4 and citrullinated CitH3 expression to reduce NET release.	Akebia Saponin D	(224)

### 6.1 Inhibition of NETs formation

#### 6.1.1 PAD4 inhibitors

PAD4 is a crucial enzyme in the synthesis of NETs. It catalyzes histone citrullination, leading to chromatin decondensation and promoting the release of nuclear DNA (233). In the context of ICH, PAD4 expression is significantly upregulated, particularly in neutrophils surrounding the hematoma. PAD4 activation promotes NETs release, thereby exacerbating neuroinflammation, BBB disruption, and vascular reperfusion impairment. Conversely, PAD4^-^/^-^ mice exhibit reduced levels of citrullinated histone H3 and attenuated BBB damage (102). Therefore, targeting PAD4 has emerged as a promising strategy to inhibit NETs formation and mitigate secondary injury following ICH.

Studies have shown that the enzymatic activity of PAD4 is significantly enhanced upon binding to Ca²^+^. Irreversible PAD4 inhibitors targeting this calcium-dependent mechanism, such as Cl-amidine and F-amidine, effectively suppress NETs release (243, 244). GSK484, a selective and reversible PAD4 inhibitor, has been shown to effectively inhibit NET formation *in vitro* (122). In SAH models, GSK484 alleviates secondary brain edema and neuronal injury by suppressing inflammation (186), and also inhibits thrombosis by blocking NETs formation (234).

Notably, in addition to conventional small-molecule inhibitors, novel compounds have also been shown to modulate PAD4 activity. Studies have shown that miR-155 enhances PMA-induced PAD4 mRNA expression and promotes NETs formation, whereas its antagonist, antagomiR-155, reduces PAD4 expression, thereby attenuating NETs release and tissue damage (204). Collectively, these findings confirm that PAD4 gene knockout or pharmacological inhibition reduces NETs formation and alleviates secondary neural injury.

#### 6.1.2 NOX and ROS inhibitors

ROS are key drivers of NETs formation, with NOX being the primary source of ROS production. Following ICH, neutrophils rapidly infiltrate the lesion site. Activation of NOX and the resulting ROS burst not only drive NETs formation but also directly contribute to BBB disruption, neuronal apoptosis, and intensified local oxidative stress. Therefore, regulating the production of ROS and NOX is critical for inhibiting NETs formation.

As a classic natural antioxidant, vitamin C not only scavenges ROS but also crosses the BBB in the form of dehydroascorbic acid (235), exerting neuroprotective effects (205, 245). Multiple clinical and preclinical studies have demonstrated that increased plasma vitamin C levels are significantly associated with a correlated risk of stroke (206, 246, 247). In animal models, vitamin C suppresses NETs formation and alleviates central inflammation by reducing ROS production (235, 245). Additionally, PKC has been shown to regulate NETosis by blocking ROS production in upstream signaling pathways (248, 249). The natural polyphenolic antioxidant curcumin exerts neuroprotective and anti-inflammatory effects by activating the Nrf2 pathway and inhibiting ROS-mediated NETs release (208).

As a key enzyme in ROS production, NOX has attracted increasing attention in NETs-targeted therapies. Diphenyleneiodonium, a non-specific NOX inhibitor, significantly reduces NETosis levels (236). In stroke models, attenuates MMP-2 and MMP-9 activity in brain tissue, alleviates cerebral edema, and mitigates BBB disruption (250, 251). Activation of NOX2 has been identified as a key mediator of NETosis and secondary brain injury. The use of NOX inhibitors has also shown beneficial effects in regulating vascular reperfusion and promoting brain tissue repair. In recent years, hypertonic saline has emerged as a promising non-pharmacological intervention with the potential to modulate NETosis. By altering the extracellular osmotic environment, it inhibits PMA- and LPS-induced ROS production, thereby interfering with both NOX-dependent and NOX-independent NETosis and promoting neutrophil apoptosis (252). This mechanism not only suppresses tissue damage caused by excessive NETs release but also contributes to the restoration of immune homeostasis.

In summary, interventions targeting the NOX/ROS pathway exert protective effects after ICH by reducing NETs formation, alleviating oxidative stress, stabilizing the BBB, and improving neurological function. These multifaceted benefits provide a strong pharmacological basis and translational potential for NETs-related therapies in ICH.

### 6.2 Promotion of NETs clearance

Current research on NETs formation has made considerable progress; however, most strategies target the early stages of NETs generation and remain insufficient to fully control NETs-mediated chronic inflammation and tissue damage. Therefore, further exploration of mechanisms for clearing established NETs may offer novel therapeutic approaches for targeting NETs-related pathological processes in central nervous system disorders.

#### 6.2.1 NE and MPO inhibitors

NE and MPO are not only essential components of NETs but also play critical roles in the inflammatory response (8, 152). Inhibiting their enzymatic activity can effectively mitigate NETs-mediated inflammation. In stroke therapy, sivelestat sodium, a commonly used NE inhibitor, has been extensively studied and applied in clinical practice. Additionally, compounds such as trigonelline, isorhamnetin, and eupafolin have also been identified as effective inhibitors of MPO (237). Studies have shown that thiocyanate, selenocyanate, and various nitrogen oxides can act as alternative substrates for MPO and directly scavenge hypochlorous acid released by neutrophils, thereby inhibiting the formation of NETs (238). However, although these inhibitors have shown some efficacy in certain inflammatory conditions, research on the protective effects of NE and MPO inhibitors in ICH remains limited. Based on current research progress, the development of inhibitory strategies targeting NE and MPO may represent a promising direction for future therapeutic interventions in ICH.

#### 6.2.2 Histone inhibitors

Histones exhibit significant cytotoxicity and, when bound to DNA, form histone-DNA complexes that constitute the structural backbone of NETs. Blocking histone-DNA complexes with monoclonal antibodies effectively inhibits NETs formation and reduces neuronal death. A study by Xu Jun et al. demonstrated that activated protein C can cleave histones and neutralize their cytotoxicity (169). Thrombomodulin-α promotes the generation of activated protein C *in vitro*, thereby reducing histone-induced thrombin production and endothelial cell death. A study by Simona Grasso et al. found that factor VII activating protease degrades histones, suppresses their cytotoxic effects on endothelial cells, reduces NETs formation, and mitigates NETosis-associated tissue damage (239).

#### 6.2.3 DNases

DNase I is frequently employed as the principal enzyme for the degradation of NETs in both animal models and *in vitro* studies (136). Its mechanism of action involves specifically recognizing and hydrolyzing extracellular double-stranded DNA and associated nucleoproteins, thereby dismantling the NETs structure. This process not only suppresses NETs formation but also attenuates NETs-induced disruption of the BBB (253, 254). DNase I is naturally abundant in the bloodstream and can continuously eliminate circulating NETs (240). Research indicates that DNase I-mediated degradation of NETs enhances the efficacy of tissue-type plasminogen activator in hematoma clearance. In animal models of ICH, combined administration of DNase I and tissue-type plasminogen activator significantly reduces cerebral edema, decreases neuronal death, and improves neurological recovery (198).

Notably, the monoclonal antibody 2C5 specifically recognizes NETs and may serve as a targeting ligand for diagnostic and therapeutic applications (255). Nina Filipczak and colleagues further conjugated the monoclonal antibody 2C5 to functionalized DNase I-loaded nanomicelles, enabling specific recognition and targeted degradation of NETs (256), thereby significantly enhancing therapeutic efficacy. Multiple animal studies have demonstrated that DNase I inhibits the cGAS-STING pathway. This inhibition reduces NETs-mediated immune activation, alleviates secondary inflammatory responses after ICH, improves BBB integrity, and mitigates neuronal injury (102, 257). These findings highlight its strong potential for neuroprotection and ICH treatment.

To date, no clinical interventions have directly addressed the role of NETs in ICH. Nevertheless, clinical trials of DNase I have been registered in patients with ischemic stroke and have advanced to early exploratory phases (NCT05203224, NCT05880524) (254). Meanwhile, animal studies indicate that DNase I confers neuroprotective effects without increasing the risk of hemorrhage. In addition, systematic reviews and clinical data demonstrate substantial NETs formation in the peripheral blood of patients with TBI, with levels closely correlating with injury severity. These findings further support the critical role of NETs in secondary inflammation following acute brain injury (258). Although DNase I has not yet been clinically applied in intracerebral hemorrhage (ICH), its use as a NET-degrading strategy has demonstrated favorable safety and partial efficacy in clinical studies of cystic fibrosis (259), COVID-19–related ARDS (241), and autoimmune diseases (242). These cross-disease clinical findings, together with preclinical results from the stroke field, collectively support the feasibility of DNase I as a NET-targeted therapy for ICH.

### 6.3 Other approaches

Studies have shown that actin and associated cytoskeletal proteins are pivotal in regulating the formation of NETs. NETs extrusion is accompanied by local cortical F-actin depolymerization, a process primarily driven by F-actin oxidation mediated by the monooxygenase MICAL-1 and facilitated by the cooperative action of G-actin, binding proteins and gelsolin (260). Myosin accumulates with cortical F-actin at the cell periphery, where actomyosin interactions generate mechanical forces that facilitate the release of NETs (261). Inhibition of MICAL-1 oxidative activity or suppression of myosin ATPase activity significantly reduces NETs formation. Additionally, variations in gelsolin levels within neutrophils influence the structural type of NETs, suggesting that cytoskeletal composition not only regulates the efficiency of NETs release but also determines their morphological characteristics (262). Metzler et al. found that inhibition of actin dynamics impairs the nuclear translocation of neutrophil elastase, thereby disrupting the formation of NETs (259).

Factor H (FH) is a principal regulator of the alternative complement pathway. Studies have shown that FH not only modulates C3b degradation by colocalizing with CD11b on the neutrophil surface but also plays multiple roles in regulating neutrophil function. Soluble factor H promotes neutrophil migration, whereas immobilized FH induces cell spreading and enhances the release of IL-8. Although factor H alone does not induce NET formation, immobilized FH significantly suppresses NET release and associated ROS production under stimulation with PMA or fibronectin combined with β-glucan, potentially attenuating local inflammation and tissue damage (263).

In addition, akebia saponin D, a compound isolated from traditional Chinese medicine, has been shown to reduce NETs release following ICH by activating the NTSR1/cAMP/PKAc/p-CREB signaling pathway and suppressing the expression of the key NETs enzyme PAD4 and its downstream product citrullinated histone H3. This intervention effectively attenuates brain tissue damage and the release of proinflammatory cytokines (224). As a free radical scavenger, edaravone can eliminate singlet oxygen and significantly inhibit NETs formation *in vitro* (264). Its compound formulation, edaravone dexborneol, has been shown in patients with acute ischemic stroke to reduce serum NETs markers while improving blood–brain barrier integrity and neurological function (222). However, most of the supporting evidence derives from ischemic stroke, and direct clinical validation in ICH populations remains lacking. Nonetheless, these findings provide a biological rationale and indirect support for targeting NETs in ICH.

### 6.4 Challenges in clinical translation

Currently, immunomodulatory strategies for hemorrhagic stroke are being investigated at multiple levels. Clinical studies have shown that immunoregulatory agents exhibit therapeutic potential in animal models. For instance, fingolimod has been reported to improve neurological recovery in SAH models by inhibiting neutrophil adhesion to the vascular endothelium (265). Minocycline has been shown to reduce the infiltration of microglia and macrophages after ICH, alleviate cerebral edema, and downregulate the expression of TNF-α and MMP-12 (266). In addition, small molecules targeting neutrophil activation, such as the FPR1 inhibitor T-0080, can mitigate brain tissue injury by interfering with the IL-1β pathway and thereby reducing microglia-mediated recruitment of neutrophils (267, 268). In recent years, NETs have emerged as key mediators of neuroinflammation and secondary injury, representing potential therapeutic targets. NETs inhibitors, including PAD4 inhibitors, NADPH oxidase inhibitors, and recombinant DNase I, have demonstrated anti-inflammatory, neuroprotective, and BBB preserving effects in various animal models of intracerebral hemorrhage. However, their clinical translation still faces numerous challenges.

First, the limited permeability of the BBB is one of the primary obstacles. Although localized BBB disruption occurs in the early phase after ICH, overall permeability remains low. Moreover, BBB integrity exhibits high temporal variability across different time windows, substantially affecting both the efficacy and safety of therapeutic agents (269, 270). Second, the formation of NETs exhibits marked temporal dependence, yet studies precisely defining their peak formation and optimal intervention window are lacking, adding to the complexity of treatment and intervention strategies (271). Further complicating the issue, the role of NETs remains controversial due to their potential dual effects. On the one hand, studies have suggested that NETs can, under certain pathological conditions, clear hemorrhage-associated debris and limit microbial invasion, thereby exerting protective effects (127). On the other hand, animal studies have demonstrated that NETs can disrupt the BBB by activating pathways such as MMP9 and AQP4, leading to cerebral edema and secondary injury (66), and may even contribute to hydrocephalus formation by impairing lymphatic clearance of cerebrospinal fluid (66). This inconsistency poses significant challenges for NET-targeted therapies, further complicating their clinical application.

Notably, most current studies on NETs remain confined to *in vitro* experiments or animal models, with limited support from clinical trials. To advance the clinical translation of NET-targeted strategies, several emerging technologies in recent years have offered new opportunities for mechanistic elucidation and target validation. Single-cell RNA sequencing can be employed to characterize the dynamic expression of NET-related genes across distinct immune cell subsets after ICH (190). Advances in *in vivo* NET imaging techniques have also provided real-time assessment tools for targeted intervention strategies (272). These cutting-edge approaches hold promise for advancing the clinical translation of NET-related therapies. However, the current challenges remain major barriers to bridging the gap between basic research and clinical application, particularly given that most studies have yet to progress to the clinical stage. Future efforts should focus on overcoming these obstacles.

## 7 Perspectives and conclusion

Secondary inflammatory responses following ICH are a key mechanism driving ongoing brain tissue damage and functional impairment. Central nervous system injury signals to the periphery via the autonomic nervous system, neuroendocrine pathways, and meningeal lymphatic vessels. This activates the peripheral immune system and promotes the infiltration of neutrophils and other immune cells into the CNS through a disrupted BBB or cerebrospinal fluid pathways. Among these processes, the bidirectional interaction between NETs and microglia plays a central role in amplifying immune responses, disrupting the BBB, and contributing to neuronal injury. Central nervous system injury also signals to the periphery via the autonomic nervous system, neuroendocrine pathways, and meningeal lymphatic vessels. These signals activate the peripheral immune system and promote the infiltration of neutrophils and other immune cells into the CNS through a disrupted BBB or cerebrospinal fluid pathways. Through the release of inflammatory mediators, coordinated activation of signaling pathways, and indirect modulation of astrocytes and other immune effector cells, NETs and microglia establish a self-amplifying and reciprocal network of immune-mediated damage that not only exacerbates brain injury but also impedes post-stroke cognitive recovery. Therefore, the NETs-microglia axis may represent a critical therapeutic target for secondary neuroinflammation following ICH, with substantial potential for clinical translation.

Although basic research has made notable progress in NETs-targeted therapies, clinical translation still faces three major challenges. First, limited BBB permeability hampers the effective delivery of most NETs inhibitors or degrading enzymes to the lesion sites within the CNS, thereby reducing their therapeutic efficacy. Second, immune defense functions must be balanced, as NETs play a vital physiological role in host antimicrobial defense. Excessive inhibition may increase the risk of infection or even promote tumorigenesis. Third, the optimal therapeutic time window remains unclear. The function of NETs varies dynamically across different phases of intracerebral hemorrhage, and the lack of systematic research to define the best intervention timing introduces additional risks and uncertainties.

Future research should focus on stratified identification of NETs-related pathways and improved precision in target selection. It is essential to develop therapeutic strategies capable of crossing the BBB and maintaining immune homeostasis while enabling controllable modulation of central inflammatory injury. Such approaches may offer viable solutions for the precision treatment of secondary neurological damage following intracerebral hemorrhage. Additionally, further exploration of the interaction between NETs and microglia in animal models of aging or common comorbidities, such as metabolic and cardiovascular diseases, is needed. This will help systematically investigate their impact on inflammation progression and neurological recovery, enhancing the translational potential of the research findings.

## Supplementary material

SUPPLEMENTARY IMAGE 1 Mechanistic illustration of the crosstalk between peripheral NETs and microglia after intracerebral hemorrhage. Proposed mechanism after intracerebral hemorrhage, showing the crosstalk between peripheral neutrophil extracellular traps (NETs) and microglia, their role in inflammatory signaling, and potential therapeutic strategies targeting NETs.
