# Supplementary figures and images for: Unveiling the inflammatory messengers after intracerebral hemorrhage: the crosstalk between peripheral NETs and microglia
Source: Front Immunol. 2025 Sep 17;16:1643524. doi: 10.3389/fimmu.2025.1643524 (PMC12483875; doi:10.3389/fimmu.2025.1643524)

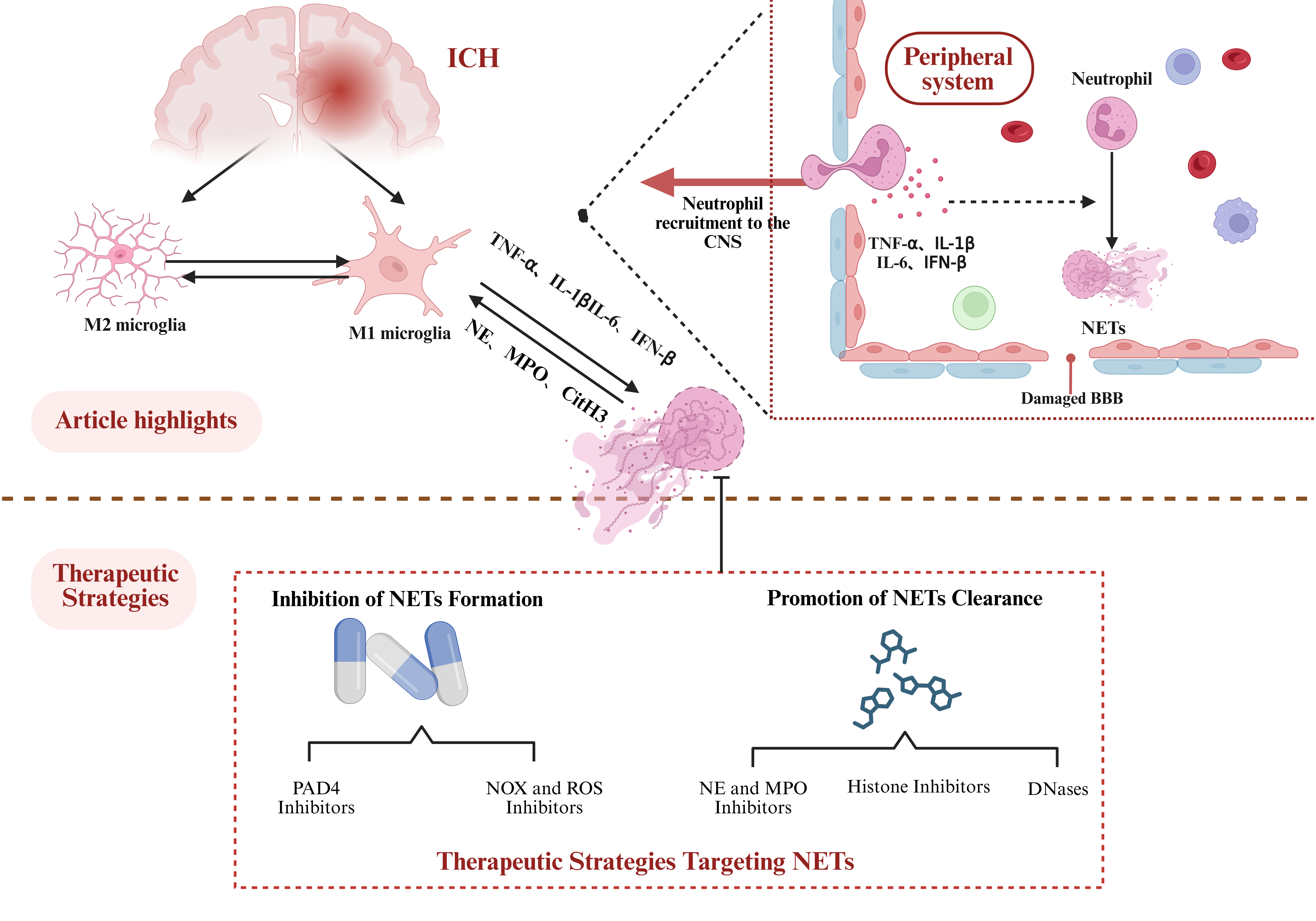

Supplement: SUPPLEMENTARY IMAGE 1 — Mechanistic illustration of the crosstalk between peripheral NETs and microglia after intracerebral hemorrhage. Proposed mechanism after intracerebral hemorrhage, showing the crosstalk between peripheral neutrophil extracellular traps (NETs) and microglia, their role in inflammatory signaling, and potential therapeutic strategies targeting NETs. [file Image1.jpeg]
